# Supplementary material for: Health-related quality of life variations by sociodemographic factors and chronic conditions in three metropolitan cities of South Asia: the CARRS study
Source: BMJ Open. 2017 Oct 15;7(10):e018424. doi: 10.1136/bmjopen-2017-018424 (PMC5652573; doi:10.1136/bmjopen-2017-018424)
Supplement: Supplementary file 1 [file bmjopen-2017-018424supp001.pdf]

Web-appendix (supplementary file)

Appendix 1: The distribution of the respondents EQ-VAS values

EQ-VAS score	Number	Percentage (%)	Cum. Percentage (%)
10-	29	0.18	0.18
20-	72	0.44	0.62
30-	88	0.54	1.16
40-	365	2.24	3.4
50-	2,056	12.62	16.03
60-	1,979	12.15	28.18
70-	3,728	22.89	51.07
80-	4,026	24.72	75.78
90-	2,594	15.93	91.71
100-	1,350	8.29	100

Notes: EQ-VAS: European Quality of Life – Visual Analogue Scale, Cum. Percentage – Cumulative percentage

Appendix 2: Relationship between EQ-VAS and EQ5D across major sub-groups

Dependent variable	Mobility	Self-care	Usual care	Pain / discomfort	Anxiety / Depression	Observation
Overall	-10.5 (0.6)	-9.9 (0.9)	-12.4 (0.7)	-10.9 (0.6)	-8.9 (0.8)	16287
Gender						
**Male**	-12.0 (0.6)	-11.4 (1.1)	-14.6 (0.9)	-11.9 (0.6)	-12.1 (0.7)	7760
**Female**	-8.1 (0.4)	-7.9 (0.7)	-9.9 (0.6)	-7.7 (0.4)	-9.1 (0.5)	8527
Age groups						
**Young (20-44 yrs)**	-9.9 (0.5)	-11.0 (0.9)	-12.0 (0.8)	-8.8 (0.5)	-9.3 (0.6)	9603
**Middle (45-60 yrs)**	-9.0 (0.5)	-10.8 (0.9)	-11.3 (0.8)	-9.0 (0.5)	-10.7 (0.6)	5544
**Elderly (>60 yrs)**	-11.1 (1.0)	-11.7 (1.6)	-13.1 (1.3)	-11.0 (1.1)	-13.2 (1.4)	1140
Income						
**Low income group (INR <10000 or US$ 155)**	-10.6 (0.4)	-12.1 (0.7)	-13.2 (0.6)	-10.2 (0.4)	-10.7 (0.5)	11537
**Middle income group (INR 10000-20000 or US$ 155-310)**	-10.2 (0.8)	-8.9 (1.6)	-10.7 (1.3)	-9.2 (0.8)	-11.8 (1.1)	2667
**High income group (INR >20000 or US$>310)**	-12 (1.0)	-14.4(2.5)	-15.6 (1.7)	-10.5 (0.9)	-8.2 (1.6)	1975
Level of education						
**Up to primary school**	-10.8 (0.6)	-12.8 (1.1)	-12.6 (0.8)	-9.9 (0.6)	-11.6 (0.8)	3604
**Secondary school**	-9.9 (0.4)	-11.1 (0.8)	-12.2 (0.7)	-9.3 (0.4)	-10.2 (0.5)	9924
**Graduation and above**	-8.9 (1.0)	-8.3 (2.1)	-11.4 (1.8)	-9.8 (0.9)	-8.3 (1.3)	2759
Marital status						
**Single**	-8.4 (1.9)	-9.4 (3.8)	-15.2 (3.3)	-9.0 (1.8)	-10.8 (2.0)	1177
**Married**	-10.3 (0.4)	-11.5 (0.6)	-12.3 (0.5)	-9.5 (0.4)	-10.3 (0.5)	14217
**Widowed**	-9.9 (1.2)	-11.1 (1.8)	-12.9 (1.4)	-10.1 (1.2)	-11.8 (1.5)	838
**Divorce**	-18.6 (5.8)	-14.1 (7.0)	-24.2 (6.0)	-15.1 (5.6)	-15.6 (5.1)	55
Tobacco use						
**No**	-10.1 (0.4)	-11.4 (0.6)	-12.4 (0.5)	-9.8 (0.4)	-10.5 (0.5)	12529
**Yes**	-13.4 (0.8)	-15.5 (1.5)	-16.5 (1.2)	-11.1 (0.7)	-12.6 (0.9)	3758
Alcohol use						
**No**	-10.6 (0.4)	-12.1 (0.6)	-13.1 (0.5)	-10.2 (0.4)	-11.0 (0.5)	13911
**Yes**	-10.6 (1.1)	-10.7 (2.0)	-11.4 (1.9)	-8.2 (1.0)	-10.6 (1.2)	2376
BMI						
**Underweight**	-10.6 (2.4)	-15.6 (5.1)	-15.4 (3.9)	-10.5 (2.9)	-7.8 (4.8)	756
**Normal weight**	-10.6 (0.9)	-11.6(1.5)	-13.6 (1.2)	-9.2 (0.9)	-9.7 (1.2)	5278
**Overweight**	-10.3 (1.1)	-12.1 (1.2)	-11.6 (1.3)	-7.7 (0.9))	-8.9 (1.2)	4190
**Obesity**	-7.4 (0.9)	-9.9 (1.7)	-11.4 (1.3)	-8.0 (0.9)	-8.6 (1.4)	2249
Diabetes						
**Diabetes (diagnosed or self-reported)**	-10.0 (0.6)	-11.6 (1.1)	-11.6 (0.9)	-9.7 (0.6)	-11.4 (0.8)	3676
**Pre diabetes**	-10.2 (0.6)	-13.5 (1.0)	-14.1 (0.8)	-9.4 (0.6)	-10.6 (0.7)	5449
**No diabetes**	-10.9 (0.7)	-11.0 (1.2)	-12.6 (1.1)	-9.9 (0.7)	-10.0 (0.8)	4610
Hypertension						

**Diagnosed or Self-reported hypertension**	-11.2 (0.6)	-14.7 (1.0)	-14.1 (0.8)	-10.8 (0.6)	-10.8 (0.7)	5074
**Pre hypertension**	-10.9 (0.7)	-12.4 (1.2)	-14.1 (1.0)	-9.7 (0.7)	-11.2 (0.9)	4717
**No hypertension**	-9.3 (0.6)	-9.6 (1.0)	-10.4 (0.9)	-8.4 (0.6)	-10.0 (0.7)	5695
Heart disease						
**No**	-10.5 (0.3)	-11.9 (0.6)	-13.0 (0.5)	-9.9 (0.3)	-10.7 (0.4)	15842
**Yes**	-9.0 (1.6)	-9.0 (2.5)	-9.5 (1.9)	-7.4 (1.7)	-11.8 (2.0)	445
Stroke						
**No**	-10.7 (0.3)	-11.9 (0.6)	-13.1 (0.5)	-10.0 (0.3)	-10.8 (0.4)	16203
**Yes**	-13.7 (3.6)	-18.4 (4.4)	-19.2 (4.1)	-12.2 (3.5)	-16.3 (4.1)	84
Kidney disease						
**No**	-10.7 (0.3)	-11.9 (0.6)	-13.1 (0.5)	-9.9 (0.3)	-10.8 (0.4)	16175
**Yes**	-11.6 (3.7)	-21.2 (4.8)	-14.3 (4.2)	-16.4 (3.2)	-16.3 (3.7)	112

NOTES: Tobacco use, Heart disease, Kidney disease were based on self-reports, newly diagnosed diabetes - defined as no self-reported diabetes and fasting blood glucose (FBG) of ≥126 mg/dl, or HbA1c ≥6.5%), pre-diabetes – no self-reported diabetes and FBG ≥100-125 mg/dl or HbA1c ≥5.7-6.4%), normoglycemia – no self-reported diabetes and FBG<100 mg/dl and HbA1c<5.7%, Newly diagnosed hypertension – defined as no self-reported hypertension and BP ≥140/90mmHg, prehypertension - no self-reported hypertension and BP: 120-139 / 80-89 mmHg and normotensive - no history of hypertension and BP <120/80 mmHg. INR: Indian rupees, mmHg – millimeter of mercury, mg/dl - Milligram/deciliter; yrs – years; US$ - United States Dollar

Appendix 3 - The comparison of HRQOL as measured by EQ5D-VAS among different countries

**Countries, year of study**	**N**	**EQ-VAS**	**Percentages with any difficulties in EQ5D domain**					
		Mean	Mobility	Self-care	Usual activities	Pain / Discomfort	Anxiety / Depression	Any dimension
India, **Delhi (age≥20), 2011**	5,365	78.9	14.1	1.6	4.4	8.0	9.5	27.8
India, **Chennai, (age≥20), 2011**	6,903	70.8	17.3	7.8	7.7	9.0	8.9	29.7
Pakistan **(Karachi), (age≥20), 2011**	4,016	73.2	10.4	1.9	5.6	8.7	9.3	17.9
China **(age≥18), 2008**	2,991	77.0	4.9	2.0	3.3	18.0	6.1	22.4
UK **(age≥18), 1998**	3395	82.5	18.4	4.2	16.3	33.0	20.9	43.1
USA **(age≥18) , 1998**	427	82.2	14.0	3.0	14.0	40.0	24.0	na
Japan **(age≥20) , 1998**	620	77.8	7.2	1.8	5.2	20.0	8.5	25.0
Spain **(age≥15) , 1998**	12,245	71.1	11.2	2.0	6.9	26.3	12.5	33.0
Canada **(age≥18), 1997**	1518	78.7	22.2	4.0	19.1	43.6	28.6	53.0
Sweden **(age≥18), 1998**	3069	83.5	10.0	2.0	8.0	42.0	30.0	na
Finland **(age≥18), 1992**	2411	79.4	20.0	5.0	18.0	39.0	14.0	na
Germany **(age≥18), 1998**	337	82.2	18.0	3.0	13.0	37.0	18.0	na
Belgium **(age≥18), 2001**	1274	81.0	13.0	3.0	15.0	42.0	21.0	na
New Zealand **(age≥18), 1999**	1328	81.3	17.0	4.0	18.0	37.0	20.0	na
Zimbabwe **(age≥18), 2000**	2350	76.1	20.0	7.0	18.0	41.0	40.0	na
Armenia **(age≥18), 2002**	2222	66.6	26.0	13.0	28.0	64.0	52.0	na

NOTES: EQ5D-VAS: European Quality of Life 5 Dimension – Visual Analogue Scale; na – not available
