# Supplementary material for: Health-related quality of life variations by sociodemographic factors and chronic conditions in three metropolitan cities of South Asia: the CARRS study
Source: BMJ Open. 2017 Oct 15;7(10):e018424. doi: 10.1136/bmjopen-2017-018424 (PMC5652573; doi:10.1136/bmjopen-2017-018424)
Supplement: Author's manuscript [file bmjopen-2017-018424.draft_revisions.pdf]

BMJ Open

BMJ Open is committed to open peer review. As part of this commitment we make the peer review history of every article we publish publicly available.

When an article is published we post the peer reviewers' comments and the authors' responses online. We also post the versions of the paper that were used during peer review. These are the versions that the peer review comments apply to.

The versions of the paper that follow are the versions that were submitted during the peer review process. They are not the versions of record or the final published versions. They should not be cited or distributed as the published version of this manuscript.

BMJ Open is an open access journal and the full, final, typeset and author-corrected version of record of the manuscript is available on our site with no access controls, subscription charges or pay-per-view fees (http://bmjopen.bmj.com).

If you have any questions on BMJ Open's open peer review process please email editorial.bmjopen@bmj.com

Health-related Quality of Life Variations by Socio-demographic Factors and Chronic Conditions in Urban South Asia: The CARRS Study

Journal:	*BMJ Open*
Manuscript ID	bmjopen-2017-018424
Article Type:	Research
Date Submitted by the Author:	27-Jun-2017
Complete List of Authors:	Singh, Kavita; All India Institute of Medical Sciences, Endocrinology & Metabolism<br>Kondal, Dimple; Publichealth Foundation of India<br>Shivashankar, Roopa; Public Health Foundation of India,<br>Ali, Mohammed; Emory University, Hubert Department of Global Health<br>Pradeepa, Rajendra; Madras Diabetes Research Foundation & Dr.Mohan's Diabetes Specialities Centre, Epidemiology & Diabetology<br>Vamadevan, Ajay; Centre for Chronic Disease Control<br>Mohan, V; Madras Diabetes Research Foundation<br>Kadir, Muhammad ; Aga Khan University, Karachi, Pakistan, Community Health Sciences<br>Sullivan, Mark; University of Washington School of Medicine,<br>Tandon, Nikhil; All India Institute of Medical Sciences,<br>Narayan, K; Emory University School of Public Health, Global Health<br>Prabhakaran, Dorairaj; Centre for Chronic Disease Control, ; Public Health Foundation of India,
**Primary Subject Heading**:	Public health
Secondary Subject Heading:	Epidemiology, Global health, Health policy
Keywords:	Health related Quality of Life (HRQOL), EQ5D, Chronic conditions, South Asia, Visual Analogue Scale

Title

Health-related Quality of Life Variations by Socio-demographic Factors and Chronic Conditions in Urban South Asia: The CARRS Study

For peer review only

For peer review only - http://bmjopen.bmj.com/site/about/guidelines.xhtml

Authors name, title, affiliation, address, contact details

Ms. Kavita Singh, MSc, PhD Candidate

Research Scientist, Centre for Chronic Conditions and Injuries (CCCI), Public Health Foundation of India (PHFI), and

Centre for Chronic Disease Control (CCDC), and

All India Institute of Medical Sciences, New Delhi

4th floor, Plot no. 47, Sector-44, Gurgaon - 122 002, Haryana

Office Landline - +91-124 – 4781400

Fax – +91-124-4722901, Mobile – +91-9899691150

Email: kavita@ccdcindia.org; kavita.singh@phfi.org

Dr. Dimple Kondal, MSc, PhD

Biostatistician, Centre for Chronic Conditions and Injuries,

Public Health Foundation of India

4th floor, Plot no. 47, Sector-44, Gurgaon - 122 002, Haryana

Office Landline - +91-124-4781400

Fax – +91-124-4722901, Mobile – +91-9899691150

Email: dimple.kondal@phfi.org

Dr. Roopa Shivashankar, MD, MSc

Senior Research Scientist and Associate Professor,

Centre for Chronic Conditions and Injuries,

Public Health Foundation of India, and

Centre for Chronic Disease Control

4th floor, Plot no. 47, Sector-44, Gurgaon - 122 002, Haryana

For peer review only - http://bmjopen.bmj.com/site/about/guidelines.xhtml

Office Landline - +91-0124 – 4781400

Fax – +91-0124-4722901, Mobile – +91-9899691150

Email: roopa@ccdcindia.org; roopa.shivashankar@phfi.org

Dr. Mohammed K. Ali, MBChB, MSc, MBA

Associate Professor, Hubert Department of Global

Health and Department of Epidemiology,

Rollins School of Public Health, Emory University, and

1518 Clifton Road NE, Room 7041, Atlanta, GA, 30322, USA

Phone number: +1 404 727 9776

Email: mkali@emory.edu

Dr. Rajendra Pradeepa, PhD

Head, Research Operations, Madras Diabetes Research Foundation

No.4, Conran Smith Road, Gopalapuram, Chennai 600086, India

Phone number: +91-44-43968888

Email: guhapradeepa@gmail.com

Dr. Vamadevan S Ajay, MPH, PhD

Senior Research Scientist, Centre for Chronic Conditions and Injuries, Public Health

Foundation of India, and

Centre for Chronic Disease Control

4th Floor, Plot No. 47, Sector 44, Institutional Area

Gurgaon - 122 002, Haryana

Office Landline - +91-124-4781400

Email: ajay@ccdcindia.org

For peer review only

For peer review only - http://bmjopen.bmj.com/site/about/guidelines.xhtml

Dr. Viswanathan Mohan, MD, PhD

President and Director, Madras Diabetes Research Foundation

No.4, Conran Smith Road, Gopalapuram, Chennai 600086, India

Phone number: +91-44-43968888

Email: drmohans@diabetes.ind.in

Dr. Muhammad M. Kadir, MBBS, FCPS

Professor, Aga Khan University, Department of Community Health Sciences,

Stadium Road, Karachi 74800, Pakistan

Phone number: +92 21 34930051

Email: masood.kadir@aku.edu

Dr. Mark Daniel Sullivan, MD, PhD

Professor, Psychiatry and Behavioral Sciences

Adjunct Professor, Anesthesiology and Pain Medicine, Bioethics and Humanities

Medical Director, UW Telepain

University of Washington, Box 356560, Seattle, WA 98195

Phone: +1-(206)685-3184, Fax: +1-(206) 221-5414

Email: sullimar@uw.edu

Dr. Nikhil Tandon, MD, PhD

Professor and Head, Department of Endocrinology & Metabolism, All India Institute of Medical Sciences,

Biotechnology Block, 3rd Floor, Rm #312, Ansari Nagar, New Delhi - 110 029, India

Phone: +91-9818211663

For peer review only - http://bmjopen.bmj.com/site/about/guidelines.xhtml

Email: nikhil_tandon@hotmail.com

Dr. KM Venkat Narayan, MD.

Ruth and O. C. Hubert, Professor of Global Health and Epidemiology, Rollins School of Public Health,

Emory University

1518 Clifton Road NE, Room 7041, Atlanta, GA, 30322, USA

Phone number: +1 404 727 9776

Email: KNARAYA@emory.edu

Dr. Dorairaj Prabhakaran*, MD, DM.

Vice President (Research and Policy),

Public Health Foundation of India, and

Director, Centre for Chronic Disease Control

4th floor, Plot no. 47, Sector-44, Gurgaon - 122 002, Haryana

Office Landline - 0124 – 4781400

Email: dprabhakaran@ccdcindia.org

*Corresponding author

Word count:

Abstract: 251 words

Main article: 3,568

Number of figures: 2

Number of Tables: 3

Appendix: Tables: 3

For peer review only - http://bmjopen.bmj.com/site/about/guidelines.xhtml

ABSTRACT [251 words]

Objectives: Health-related quality of life (HRQOL) is a key indicator of health. However, HRQOL data from representative populations in South Asia are lacking. This study aims to describe HRQOL overall, by age, gender, and socio-economic status (SES), and examine the associations between selected chronic conditions and HRQOL in adults from three urban cities in South Asia.

Methods: We used data from 16287 adults aged ≥20 years from the baseline survey of the Centre for Cardiometabolic Risk Reduction in South Asia (CARRS) cohort (2010-11). HRQOL was measured using the European Quality of Life 5 dimension - Visual Analogue Scale (EQ5D-VAS), which measures health status on a scale of 0 (worst health status) – 100 (best possible health status).

Results: 16,284 participants completed EQ5D-VAS. Mean age was 42.4(+/-13.3) years and 52.4% were women. 14% of the respondents reported problems in mobility, and pain/discomfort domains. Mean VAS score was 74 (95% confidence interval [CI]: 73.7, 74.2). Significantly lower health status was found in elderly (64.1), women (71.6), unemployed (68.4), less educated (71.2) and low-income group (73.4). Individuals with chronic conditions reported worse health status than those without (67.4 vs. 76.2): Odds Ratio: 1.8 [95%CI: 1.61, 2.04].

Conclusions: The mean VAS (74.5) reported in our study is much lower than developed countries (82.5). Our data demonstrate significantly lower HRQOL in key demographic groups and those with chronic conditions, which are consistent with previous studies. These data provide insights on inequalities in population health status, and potentially reveal unmet needs in the community to guide health policies.

Key words (5)

Health related Quality of Life (HRQOL), EQ5D, Chronic conditions, South Asia, Visual Analogue Scale

For peer review only - http://bmjopen.bmj.com/site/about/guidelines.xhtml

Strengths and limitations of this study

- This is the first population level health related quality of life (HRQOL) data from South Asia using EQ5D-VAS including three large metropolitan cities in India and Pakistan with a large sample size (16,284 adults aged ≥20 years).
- Our data provide the first baseline values to be used for monitoring population health status and analysed the relationships between selected chronic conditions and HRQOL.
- HRQOL data presented in this article could be used to complement national targets by providing a measure of chronic disease burden based on perceived health status rather than solely on mortality and disease prevalence.
- Due to the cross-sectional nature of the data, the causal relationship between socio-economic parameters/chronic conditions and HRQOL cannot be determined.
- Many chronic conditions (respiratory, locomotor, cancer, etc.) were not included in the survey. Therefore, the ranking of most severe health conditions and associated HRQOL is not complete.

For peer review only

For peer review only - http://bmjopen.bmj.com/site/about/guidelines.xhtml

Main article [3568 words]

Introduction

Health-related Quality of Life (HRQOL) is a multidimensional concept that provides a broader perspective of health through conveying an individual's ability to function in physical, mental, and social domains of life (1). HRQOL is thus an essential patient-centred outcome measure, which is useful to guide health policies (2, 3). HRQOL is preferred over other health indicators (life expectancy, mortality, morbidity) for measuring chronic disease burden as it incorporates both length and quality of life (4). In South Asia, chronic conditions (diabetes, hypertension and heart diseases) occur at an early age (5) with detrimental effects on length and quality of life (6, 7). In addition, episodes and fear of hypoglycaemia, recurrent heart attacks, stroke and other long-term complications (kidney diseases, diabetic retinopathy) are not always measured as such, though they have a substantial adverse impact on an individual's overall health status (8). Therefore, it is important to quantify the effect of chronic conditions on individuals HRQOL.

There are several disease-specific (Chronic respiratory distress questionnaire, Arthritis Impact Measurement Scale) and generic instruments (Short Form 36, World Health Organization (WHO) – Quality of Life questionnaire, and European Quality of Life Five Dimensions – Visual Analogue Scale (EQ5D-VAS)) available to measure population HRQOL (4, 9-18). However, the EQ5D-VAS is favoured because it is generic, not specific to a particular disease, and it not only includes multidimensional measures of health profile in five dimensions (mobility, self-care, usual activities, pain/discomfort and anxiety/depression), but also includes the single-dimensional measure VAS, to summarize overall health status (1). Also, EQ5D-VAS has been applied and validated for

For peer review only - http://bmjopen.bmj.com/site/about/guidelines.xhtml

its use in many population surveys across the world, therefore, it makes the comparison of health status across populations easier.

Data on population HRQOL across socioeconomic status (SES) from South Asia are scarce, and little is known about the relative associations between different chronic conditions and individual HRQOL. The Centre for Cardio-metabolic Risk Reduction in South Asia (CARRS) study (19) had collected data on both EQ5D-VAS and selected chronic conditions from a large representative population of adults in urban South Asia. We used this opportunity to examine population HRQOL in this region. In this paper, we describe the variations in HRQOL by age, gender, and SES, and explore the relationships between selected chronic conditions and HRQOL in a representative sample of adults aged ≥20 years from three metropolitan cities in India and Pakistan. We also analysed the relationship between multidimensional EQ5D measures and single dimensional VAS across major subgroups.

Methods

Study design and setting

We obtained data from the baseline cross-sectional survey of the CARRS cohort (2010-11), which recruited a representative sample of non-pregnant adults aged ≥20 years from three urban cities: Chennai, Delhi, and Karachi. These metropolitan cities with large and heterogeneous populations in terms of demographic profile and economic transitions offer unique opportunities to assess variations in health status across different socio-economic groups. The detailed CARRS study design has been published elsewhere (19). Briefly, a multi-stage cluster random sampling strategy was used with wards (in Delhi and Chennai) or clusters (in Karachi) as the primary sampling units. Using the WHO STEPS survey "Kish method", two participants, one male and one female,

For peer review only - http://bmjopen.bmj.com/site/about/guidelines.xhtml

aged ≥20 years (non-pregnant) and meeting the study eligibility criteria, were selected from each randomly selected household (19).

Study measures

Comprehensive and uniform data collection instruments were used to capture measurements in all three sites. A summary of all surveillance measures, methods, and instruments used in the study has been published in detail (19). Briefly, a questionnaire was administered to collect information regarding demographic, socio-economic, behavioural, and past and present health status of the participant.

Trained study staff measured anthropometric parameters (height, weight) using standardized techniques and blood pressure (BP) twice at each participant's home or at a medical camp organized in the community, after five minutes in a seated position using an electronic BP measuring device (Omron Dailan Co., China). If the difference between the first two systolic or diastolic BP readings was more than 10 mmHg or 5 mmHg, respectively, a third reading was taken. Average BP readings of the two/three readings were recorded in the study database. Additionally, fasting blood glucose and glycated haemoglobin (HbA1c) were measured. The overall response rates were 94.7% for questionnaire completion and 84.3% for blood tests.

Population health status was measured using the EQ5D-VAS questionnaire, which consisted of two components; health state description and self-rated health status on VAS. Health state description (profile) includes five dimensions (5D); mobility (walking ability), self-care (ability to wash or dress by oneself), usual activities (ability to work, study, housework), pain/discomfort, and anxiety/depression. The respondents self-rate their level of severity for each dimension using three levels (EQ5D-3L): having no problems, having some or moderate problems, or being unable to do/having extreme problems. The respondents were asked to choose one of the statements which best described their health status on the surveyed day. For example, three levels of 'mobility'

For peer review only - http://bmjopen.bmj.com/site/about/guidelines.xhtml

dimension were phrased as "I have no problems in walking," "I have some problems in walking," and "I am confined to bed". Given the possible permutations of different domains and response types, there are potentially 243 (=3^5) different health profiles.

For overall health status, the respondents evaluated their health status using the VAS. The VAS asks respondents to mark health status on the day of the interview on a scale of 0 (worst health status) – 100 (best imaginable health status).

Covariates: Self-reported age at baseline in completed years was used and categorized into 20-24, 25-34, 35-44, 45-54, 55-64, 65-74 and ≥75. Based on participant responses, we categorized employment status into employed, student, housewife, retired, and unemployed. Income class was grouped into three categories based on household monthly income: low-income (<10,000 INR), middle-income (10,000-20,000 INR) and high-income strata (>20,000 INR). We categorized highest education level attained into three categories - up to primary, secondary schooling, and graduates. The marital status was classified as: single, married, widowed, and divorced. Body mass index (Kg/m^2) of ≤18 was used to define the underweight, and >18-25: normal weight, >25-30: overweight and ≥30: obese. Lifestyle habits like tobacco use was classified based on self-reports as never, former and current user. Data on chronic conditions consisted of self-reported hypertension, diabetes, heart disease, stroke, and kidney disease. In addition, diabetes was categorized into self-reported, newly diagnosed (defined by no self-reported diabetes and fasting blood glucose (FBG) of ≥126 mg/dl, or HbA1c ≥6.5%), pre-diabetes (no self-reported diabetes and FBG ≥100-125 mg/dl or HbA1c ≥5.7-6.4%) and normoglycemia (no self-reported diabetes and FBG<100 mg/dl and HbA1c<5.7%). Similarly, we classified hypertension as: self-reported, newly diagnosed (no self-reported hypertension and BP ≥140/90mmHg), prehypertension (no self-reported hypertension and BP: 120-139 / 80-89 mmHg) and normotensive (no history of hypertension and BP <120/80 mmHg).

For peer review only - http://bmjopen.bmj.com/site/about/guidelines.xhtml

Ethical considerations

The CARRS study has obtained institutional ethics approval from each of the participating institutions: Public Health Foundation of India, All India Institute of Medical Sciences, New Delhi, Madras Diabetes Research Foundation, Chennai, Aga Khan University, Karachi and Rollins School of Public Health, Emory University, USA. Study participants provided written informed consent, before participation in the study.

Analysis

We used Stata (version 14.0 SE; StataCorp, TX, USA) for data analysis. We used the 'svy' command for all analysis to account for the complex survey design (20). Before any of the survey estimation commands were used, the svyset command was used to specify the variables that described the stratification, sampling weight, and primary sampling unit variables. This analysis included data obtained from 16,284 study participants. All the responses coded as refused, unknown, or missing were treated as missing data. The frequency (percentages) and mean were reported to display the level of population health status and the sample characteristics. Percentages of those reporting any problems in EQ5D domains and mean VAS were stratified by respondent's demographic characteristics - age, gender, marital status, and SES - education, income, and employment status; and health-related indicators - presence of chronic conditions, were reported. Additionally, odds-ratios of moderate or severe health problems in people with and without chronic conditions was analysed using logistic regression. The model was adjusted for socio-demographic covariates (age, gender, marital status, education level, and household income). Linear regression was performed to explore the relationship between the VAS and the EQ5D measures across major subgroups. In the regression model, VAS was used as a dependent variable, and EQ5D measures were treated as independent variables.

For peer review only - http://bmjopen.bmj.com/site/about/guidelines.xhtml

Study Results

Characteristics of the study population

A total of 17,274 individuals in 10,002 households were approached in the three study sites (7,596 participants in Chennai, 5,420 in Delhi, 4,258 in Karachi). From these, a total of 16,287 participants were recruited (the overall response rate was 94.3% at the participant level; 6,906 Chennai [90.9%], 5,364 Delhi [98.9%], and 4,017 Karachi [94.3%]). Detailed baseline characteristics of the CARRS cohort is published elsewhere (21-24). Briefly, mean age was 42.4 (+/- 13.3), 52.4% were females, 61% completed secondary schooling, and the majority of respondents (72.5%) reported household income level <INR. 10,000 (US$200). A third (66%) of the study population had BMI ≥25, and one-fifth (20%) of the respondents reported current tobacco use and 37.5% had self-reported chronic conditions (hypertension, diabetes, heart disease, stroke or chronic kidney disease).

Overall HRQOL by age and gender

A total of 16,284 study participants completed the EQ5D-VAS [99.9%]. Overall, the percentage of respondents reporting any problems in mobility and pain/discomfort (14% each) were higher than for other domains. Greater health problems were observed with higher age for both men and women (p-value <0.001). [Table 1]. Problems with mobility were higher with advancing age. However, problems with anxiety/depression did not show such trend. Average health status (VAS) reported by the CARRS cohort was 74.5 (95% CI 73.7 – 74.2). [Figure 1]. Women reported lower health status than men (71.6 vs.79.0).

74% of the respondents rated a perfect health profile with no difficulties in any EQ-5D domain, and 0.06% rated the worst health profile whereby they had difficulties with every EQ-5D domain. The distribution of the VAS scores was skewed in the direction of

For peer review only - http://bmjopen.bmj.com/site/about/guidelines.xhtml

best-imagined health state. Only 0.5% respondents rated their health status on VAS under 10, and 10% rated it under 50 [Appendix 1].

HRQOL and socio-economic status

Table 2 and Figure 2 depicts the mean VAS, percentage, and odds of respondents reporting any problems in the five dimensions, across various sub-groups, respectively. Employed adults and students reported better health status than homemakers, retired, or unemployed participants. We observed almost equal health status in homemakers and retired people. Health status was also similar in the middle- and high-income groups, while it was significantly lower in the low-income group. Individuals with higher education (graduate and above) and high income had higher HRQOL than those with secondary or primary schooling and low-income class. Also, individuals with BMI ≥18-24 Kg/m^2 reported better health status, than those with BMI ≥25 Kg/m^2. Current tobacco users reported better health status than former tobacco users or non-users. However, in a stratified analysis of HRQOL in tobacco users by presence or absence of chronic conditions, tobacco users with chronic conditions reported worse health status than non-users.

HRQOL and chronic conditions

Overall, individuals with chronic conditions reported lower health status than those without chronic conditions. About half of the respondents with self-reported diabetes, hypertension, stroke, heart disease, or chronic kidney disease reported moderate or severe problems in all five domains (Table 2).

Table 3 presents the adjusted odds ratio of any problems (moderate or severe) comparing people with versus without chronic conditions, stratified by sex and cities. Individuals with chronic conditions reported two times greater problems in mobility, usual activities domains, pain/discomfort, and anxiety/depression, than in individuals without chronic conditions.

For peer review only - http://bmjopen.bmj.com/site/about/guidelines.xhtml

Further, a small proportion of individuals with chronic conditions, mostly those with hypertension (10.5%) or diabetes (8.3%) reported having a perfect health state.

Relationship between VAS and EQ5D measures across major sub-groups

We expected that each EQ5D dimension would have an independent relationship with VAS since each of them represents a different aspect of HRQOL. Appendix 2 provides the beta coefficients of the weighted regression models (i.e., with the application of the population sampling weights). In the overall population, having any problems in mobility, self-care, pain/discomfort, and anxiety/depression were associated with VAS scores that were 10-12 points lower. This inverse relationship of lower VAS with higher domain difficulties was larger in men, elderly (>60 years), low-income, less educated, divorced, and high BMI individuals, compared to their respective counterparts. Tobacco users who reported difficulties in all domains of EQ5D had lower VAS scores (indicating lower quality of life). Kidney disease and stroke were the most disabling conditions on all measures.

Discussion

Comparative assessments of HRQOL variations by socio-demographic factors and chronic conditions aid in prioritizing public health targets for intervention. Results from this study indicate that less than 10% of the respondents rated their health status as 100 (i.e. best imagined health state) on VAS. Mobility, pain/discomfort, and anxiety /depression were the most commonly reported problems, with the extent of these problems differing across population subgroup. Elderly (>60 years) and women reported significantly greater problems in the mobility, pain/discomfort and anxiety/depression domains.

For peer review only - http://bmjopen.bmj.com/site/about/guidelines.xhtml

The mean VAS in our study was 74.5, which is lower than reported by most western countries (82.5), but comparable to the results from other low- and middle-income countries (LMICs) (71.1 - 77.8) [Appendix 3] (25-28). Lower health status reported by urban South Asians can be interpreted in a number of ways. The lower scores may be related to generally lower reporting of HRQOL among this group. Alternatively, these scores may reflect morbidity and sub-optimal access to healthcare facilities to address health concerns. South Asians experience chronic conditions at relatively younger ages than other race/ethnic groups and the associated reductions in productivity and income levels may be manifested in these self-reported EQ5D-VAS scores.

Mobility is the dimension that has the highest rate of having problems (14-17%) in South Asia, which is comparable to results from other LMICs (29). However, problems in anxiety/depression are pretty low in South Asia when compared to the rest of the world; this could be due to under diagnoses of depression related problems or stigma attached to these health conditions. These patterns could also reflect differences in how adults in different parts of the world self-rate their health.

Worse health status in retired or homemakers, compared to employed persons may be related to being homebound or reflect underlying illnesses that may be the factor driving these participants to be homebound and not employed.

In terms of modifiable risk factors, maintaining a healthy BMI cut-off (18-25 Kg/m^2) is favoured because, individuals with BMI <18 Kg/m^2 and ≥25 Kg/m^2, reported greater problems in all five domains. Although, previous studies have shown that lower levels (intensity) of tobacco use are linked with higher HRQOL and regular tobacco users with worse health status (30, 31), in our study, former tobacco users reported lower HRQOL than current users. This finding may indicate reverse causality, i.e. former tobacco users after experiencing an illness would have quit smoking/tobacco. Further, supported by

For peer review only - http://bmjopen.bmj.com/site/about/guidelines.xhtml

the fact that tobacco users with chronic conditions or greater difficulties in EQ5D domains had lower VAS scores is suggestive that morbidity and not the habit of tobacco use per se are more closely related to participants' perception of health. However, a causal link between tobacco use and HRQOL cannot be confirmed in this cross-sectional study. Longitudinal analyses of the independent associations between the smoking/tobacco with HRQOL may provide a better understanding of this relationship.

The lower health status reported by females, less educated, unemployed, and low-income groups may indicate higher levels of stress in these groups (17). Other potential contributing factors that are known to influence health status are living conditions, Gross domestic product (GDP) per capita, inequities in income distributions, and access to healthcare (32-37). Therefore, public health initiatives should focus on inter-sectoral approaches to address issues of education, generating more avenues for employment, and improving the quality of primary healthcare.

Notably, one in five individuals living with known hypertension or diabetes (average disease duration four years) still reported a perfect health state, indicating that these individuals may feel asymptomatic until they experience a clinical event. Also, very small proportions of patients with heart disease and stroke (with longer duration of illness; average nine years), reported perfect health states, suggesting that these individuals may have adapted to their conditions over time and maybe benefiting from treatment and self-care that improves their self-rated quality of life. However, we did not investigate whether these other factors like adherence influence quality life in those living with chronic conditions.

Due to the differences in statistical analyses, HRQOL measures, socio-demographic characteristics of the sample, and medical conditions selected, the results of this study may not be directly comparable to reports from other countries (38). Nevertheless, a

For peer review only - http://bmjopen.bmj.com/site/about/guidelines.xhtml

For peer review only

few differences and common findings are noteworthy. Individuals with stroke or chronic kidney disease rated the lowest health status, which is consistent with results reported from other studies done in China, Thailand, and Western populations (28, 39-42). Since the respondent's health status could be affected by how well the condition was managed, caution is needed in interpreting study results regarding the relative effect of chronic conditions on HRQOL (43-47). A more recent Canadian study conducted by Mo *et al*. indicated a strong relationship between low health utility index (HUI) scores and certain chronic conditions (48). The authors found that arthritis/ rheumatism, heart disease, hypertension, cataracts, and diabetes had a negative impact on HRQOL. In the US, Medical Expenditure Panel Survey data based study reported that, after adjusting for socio-demographic variables, all of the selected chronic conditions were associated with lower EQ5D scores, with effects greatest for emphysema, followed by heart disease, stroke, high BP, diabetes, and asthma(49, 50).

Lastly, the issues of 'clinical' or 'policy' relevance of the difference in EQ5D measures needs much discourse. For example, if the VAS in two groups of the population is 5 or 10 points different from each other, we will not be able to make a clinical judgment on how much these two groups would differ in their actual health status. These issues relate to determining a minimally significant difference/change in HRQOL needs investigation in future studies. Also, the findings of this study may not be replicable if researchers use a different HRQOL instrument (51-56), which can be tested in a future study.

To our knowledge, this is the first population level HRQOL data from South Asia using EQ5D-VAS including three large metropolitan cities in India and Pakistan with a large sample size that has used multistage cluster random sampling strategy and standardized protocols and measurement tools across sites. Our data provide the first baseline values to be used for monitoring population health status and analysed the

For peer review only - http://bmjopen.bmj.com/site/about/guidelines.xhtml

relationships between selected chronic conditions and HRQOL. This information could be used to complement national targets by providing a measure of chronic disease burden based on perceived health status rather than solely on mortality and disease prevalence. In our secondary data analysis, EQ5D and VAS measures correlated well, which confirms the convergent and discriminate validity of the EQ5D instrument.

There are several limitations to this study. First, due to the cross-sectional nature of the data, the causal relationship between socio-economic parameters/chronic conditions and HRQOL cannot be determined and is not implied. Second, many chronic conditions (respiratory, locomotor, cancer, etc.) were not included in the survey. Therefore, the ranking of most severe health conditions and associated HRQOL is not complete. Third, the selected chronic conditions were self-reported, and the study investigators did not examine the accuracy of information. However, this poses less of a threat to validity because self-reporting of heart diseases, stroke, and kidney diseases are pretty accurate in community surveys (57-60). Further, hypertension and diabetes were measured in this study using standardized methods. Lastly, EQ5D data were self-reported and the variation in how individuals perceive disability varies widely. However, this should be less of a problem given the large sample size in this study.

For peer review only

Conclusion

HRQOL appears to be lower with higher age and among women in South Asia. Our data demonstrates significantly lower HRQOL in key demographic groups and those with chronic conditions, which are consistent with previous studies. These data provide insights on inequalities in population health status, and potentially reveal unmet needs in the community to guide health policies.

For peer review only - http://bmjopen.bmj.com/site/about/guidelines.xhtml

Funding: The CARRS Study was funded in whole or in part by the National Heart, Lung, and Blood Institute of the National Institutes of Health, Department of Health and Human Services (contract no. HHSN268200900026C) and the United Health Group (Minneapolis, MN, USA). Roopa Shivashankar is supported by a Wellcome Trust Capacity Strengthening Strategic Award Extension phase to the Public Health Foundation of India and a consortium of UK universities (WT084754/Z/08/A).

Conflict of Interest: All authors [KS, DK, RS, MKA, RP, VSA, VM, MMK, MDS, NT KMVN, and DP) declare that they have no conflict of interest to report.

Author's contribution: KS, DP, MKA, RS, NT and KMVN conceptualized and designed the study. KS wrote the first draft of the manuscript. KS and DK performed statistical analysis. RS, VSA, MKA, RP, VM, MMK, MDS, NT, KMVN, and DP contributed significantly in the revision of the manuscript. All authors have approved the submission of this version of the manuscript.

Data sharing statement: KS, DK, and DP have access to study dataset and statistical code. Any request for data sharing should be addressed to the corresponding author (DP).

For peer review only

For peer review only - http://bmjopen.bmj.com/site/about/guidelines.xhtml

Tables

Table 1 - Percentage of respondents reporting moderate or severe problems in EQ5D domains, stratified by age and gender

EQ5D Dimensions	20-24 years	25-34 years	35-44 years	45-54 years	55-64 years	65-74 years	≥75 years	Overall
**Overall (N)**	1179	3752	4672	3539	2005	878	262	16287
Males (N)	591	1614	2128	1723	1026	500	178	7760
Females (N)	588	2138	2544	1816	979	378	84	8527
**Mobility**								
All respondents (%)	5.3	8.3	13.6	18.1	23.5	31.2	39	14.6
95% CI	[4.0,7.0]	[6.8,10.0]	[11.7,15.9]	[16.1,20.3]	[20.8,26.4]	[27.5,35.2]	[32.7,45.6]	[13.3,15.9]
Male (%)	2.9	3.6	6	8.7	17	20.5	34.7	8.2
95% CI	[1.5,5.6]	[2.6,5.0]	[4.8,7.3]	[7.2,10.4]	[14.1,20.4]	[16.7,24.9]	[27.5,42.7]	[7.3,9.2]
Female (%)	7.8	11.8	20.1	26.9	30.6	45.6	48	20.3
95% CI	[5.9,10.2]	[9.7,14.4]	[17.1,23.4]	[24.0,29.9]	[26.5,35.1]	[40.8,50.6]	[36.2,60.1]	[18.5,22.3]
**Self-care**								
All respondents (%)	1.6	2.6	3.8	4.7	6.9	9	14.6	4.2
95% CI	[1.0,2.8]	[2.0,3.5]	[2.9,5.0]	[3.8,5.7]	[5.4,8.8]	[6.9,11.8]	[10.3,20.2]	[3.6,4.9]
Male (%)	1.2	1.5	1.7	2.7	5.1	5.2	14	2.6
95% CI	[0.4,3.6]	[0.8,2.5]	[1.0,2.9]	[1.8,3.9]	[3.1,8.2]	[3.3,8.0]	[8.9,21.5]	[2.0,3.3]
Female (%)	2.1	3.5	5.5	6.5	8.9	14.2	15.6	5.6
95% CI	[1.2,3.5]	[2.5,5.0]	[4.1,7.5]	[5.2,8.2]	[6.7,11.6]	[10.6,18.7]	[9.4,24.9]	[4.6,6.8]
**Usual activities**								
All respondents (%)	2	3.5	4.8	7.1	10.7	16.6	23.1	6.0
95% CI	[1.2,3.2]	[2.8,4.4]	[3.8,5.9]	[6.0,8.4]	[8.9,12.8]	[13.9,19.7]	[17.8,29.4]	[5.4,6.8]
Male (%)	1.3	1.4	1.8	3.2	6.4	11	19.7	3.2
95% CI	[0.5,3.7]	[0.9,2.1]	[1.2,2.7]	[2.3,4.3]	[4.8,8.6]	[7.9,15.2]	[13.8,27.2]	[2.7,3.9]
Female (%)	2.6	5.1	7.2	10.7	15.4	24.1	30.3	8.5
95% CI	[1.6,4.3]	[4.0,6.6]	[5.6,9.2]	[8.8,12.9]	[12.3,19.1]	[20.4,28.1]	[20.7,42.0]	[7.4,9.7]
**Pain/Discomfort**								
All respondents (%)	6	9.2	13.4	18.6	20.4	27.1	30.3	14.3
95% CI	[4.4,8.1]	[7.7,10.9]	[11.5,15.5]	[16.6,20.7]	[17.9,23.1]	[23.3,31.2]	[24.1,37.2]	[13.0,15.6]
Male (%)	3.6	5.5	7.6	10.6	14.1	16.5	28	8.9
95% CI	[2.3,5.7]	[4.0,7.3]	[6.0,9.6]	[9.1,12.4]	[11.4,17.4]	[13.1,20.7]	[20.7,36.7]	[8.0,9.9]
Female (%)	8.5	11.9	18.2	25.9	27.2	41.2	35.1	19.1
95% CI	[5.7,12.3]	[9.8,14.5]	[15.5,21.3]	[23.0,29.1]	[23.5,31.1]	[35.6,47.0]	[25.0,46.7]	[17.1,21.2]
**Anxiety/Depression**								
All respondents (%)	4.9	5.8	7.6	9.9	10.9	13.7	18	8.1
95% CI	[3.7,6.6]	[4.8,7.0]	[6.5,8.9]	[8.7,11.4]	[9.4,12.7]	[11.2,16.7]	[13.3,23.7]	[7.4,8.9]
Male (%)	3.7	4.1	5.4	6.7	8.7	7.6	15.7	5.9
95% CI	[2.2,6.0]	[3.1,5.5]	[4.3,6.8]	[5.2,8.5]	[6.8,11.0]	[5.4,10.7]	[10.7,22.6]	[5.2,6.7]
Female (%)	6.2	7.0	9.5	13	13.4	21.9	22.7	10.1
95% CI	[4.4,8.6]	[5.6,8.8]	[7.9,11.4]	[11.0,15.2]	[11.1,16.1]	[18.0,26.5]	[14.0,34.6]	[9.1,11.3]

Abbreviations: CI – confidence interval, EQ5D – European Quality of Life 5 dimension, N – number of participants

For peer review only - http://bmjopen.bmj.com/site/about/guidelines.xhtml

Figure 1 - Mean self-rated health status using EQ5D-VAS of respondents by age groups and gender

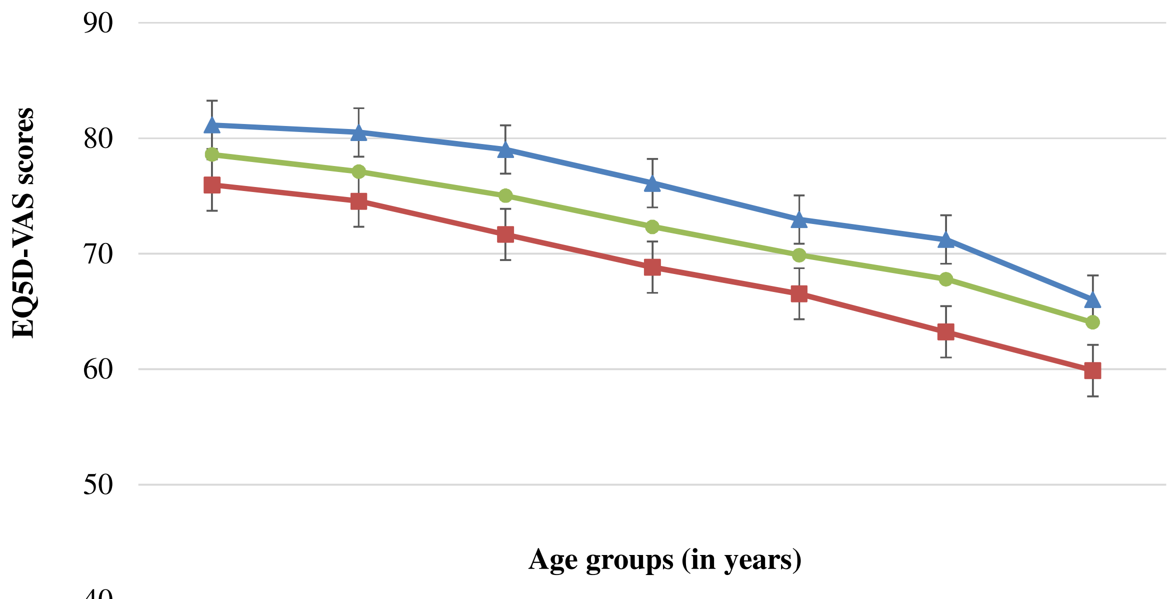

	20-24	25-34	35-44	45-54	55-64	65-74	>75
Males	81.15	80.51	79.02	76.11	72.95	71.22	66.02
Females	75.95	74.55	71.66	68.83	66.53	63.23	59.88
Total	78.6	77.11	75.03	72.33	69.88	67.8	64.06

Figure Legend: This figure presents the mean self-rated health status for overall study population by age-groups and gender. European Quality of Life 5 Dimension - Visual Analogue Scale (EQ5D-VAS) measures health status on a scale of 0 (worst health status) – 100 (best imaginable health status)

For peer review only - http://bmjopen.bmj.com/site/about/guidelines.xhtml

Figure 2 - Odds of moderate or severe health problems by socio-demographic factors and chronic conditions

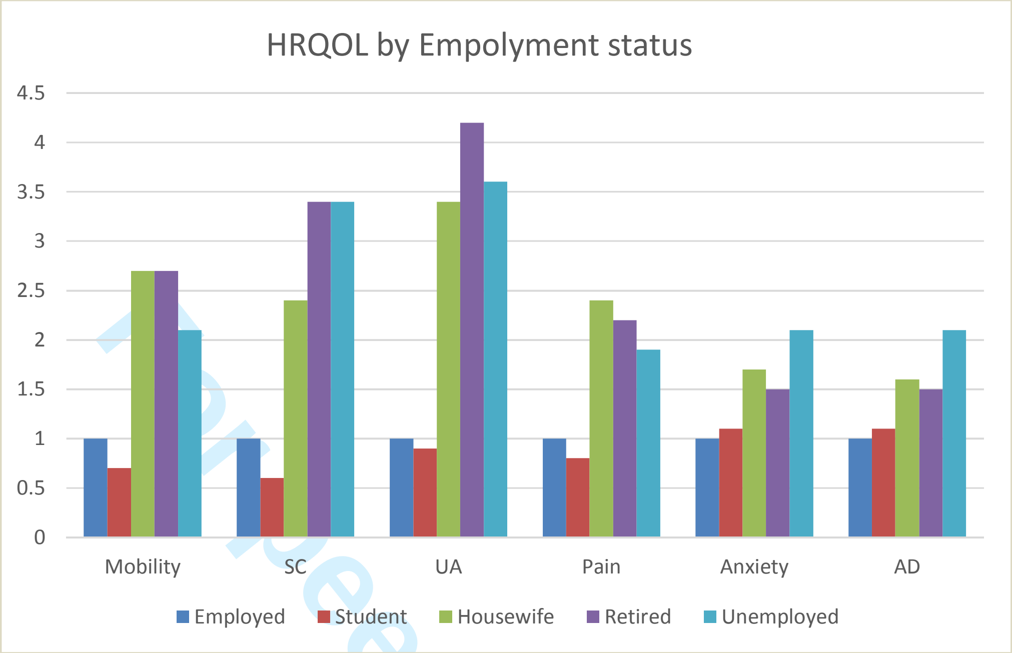

SC – Self-Care, UA – Usual Activities, AD – Any Dimension

Figure 2.a. shows the odds of moderate or severe difficulties in EQ5D domains (mobility, self-care, usual activities, pain/discomfort, anxiety/depression and any of the five dimensions) by employment status. With reference to those who were employed (OR=1), housewife, retired, and unemployed reported greater problems in all five domains. Whereas, students only reported higher anxiety problems compared to employed.

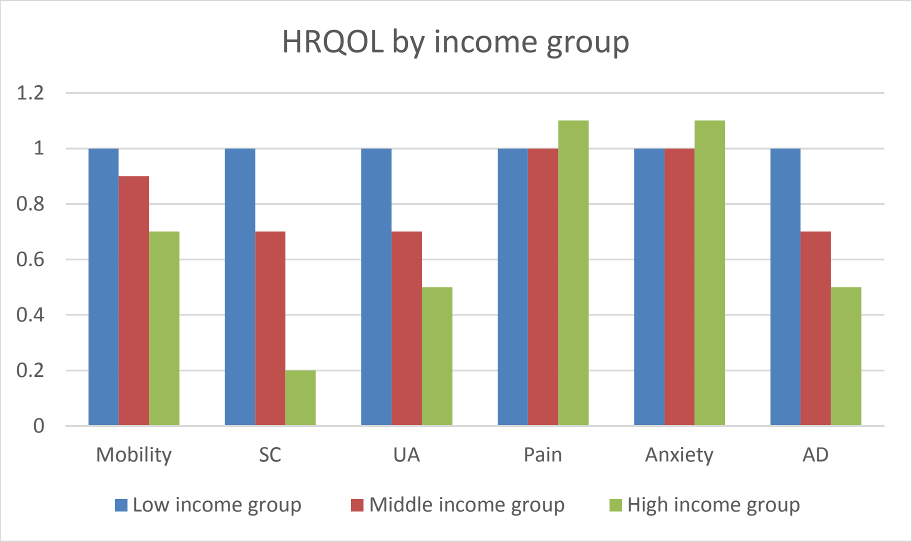

Figure 2.b. shows the odds of moderate or severe difficulties in EQ5D domains by income-group. With reference to low-income group (OR=1), those in middle- or high- income groups had less problems in all five domains.

For peer review only - http://bmjopen.bmj.com/site/about/guidelines.xhtml

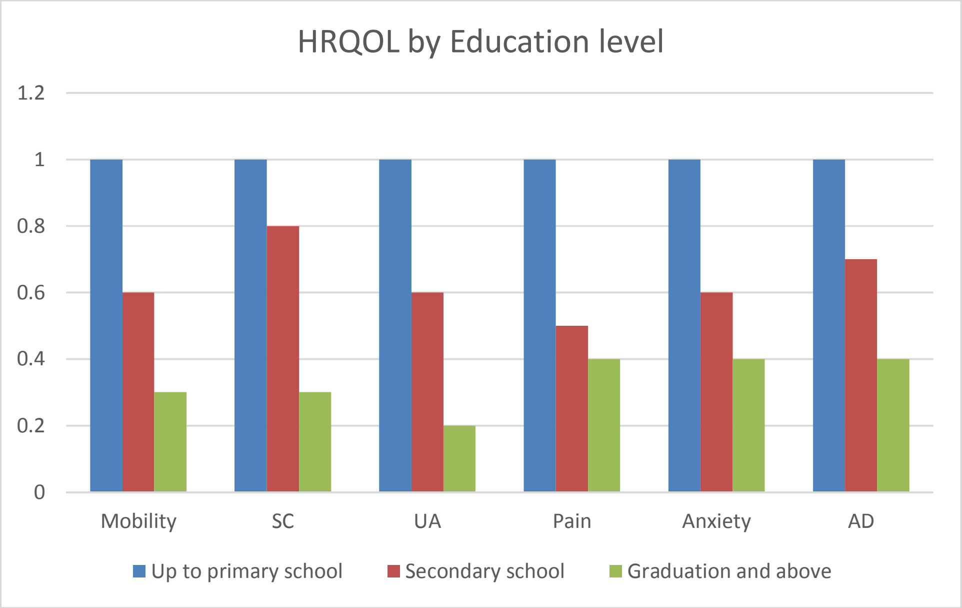

Figure 2.c. shows the odds of moderate or severe difficulties in EQ5D domains (mobility, self-care, usual activities, pain/discomfort, anxiety/depression and any of the five dimensions) by education level. With reference to those primary school education (OR=1), individuals with secondary school or graduates reported significantly lower problems in all five domains.

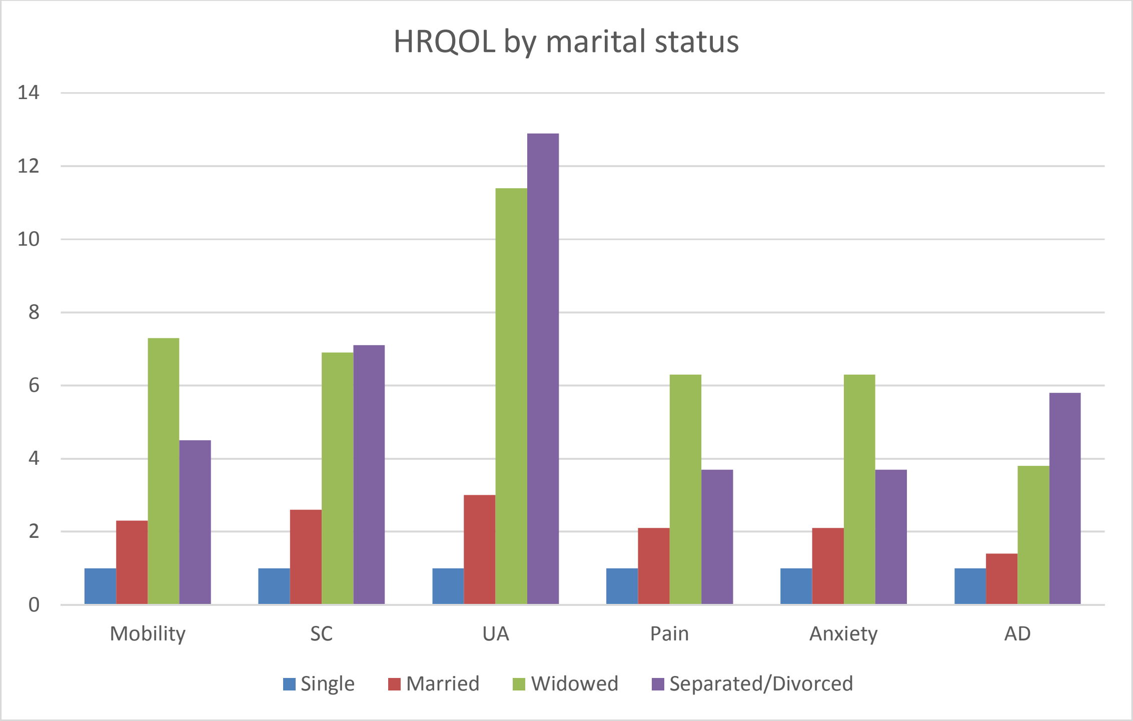

Figure 2.d. shows the odds of moderate or severe difficulties in EQ5D domains by marital status. With reference to single (OR=1), those who were married, widower, or divorcee had greater problems in all five domains.

For peer review only - http://bmjopen.bmj.com/site/about/guidelines.xhtml

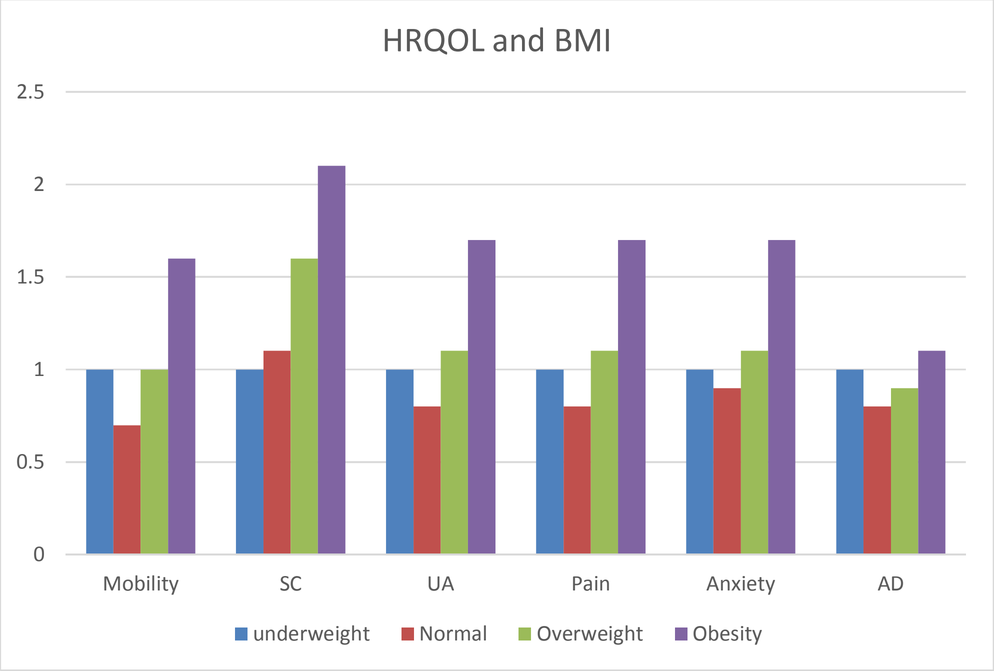

Figure 2.e. shows the odds of moderate or severe difficulties in EQ5D domains (mobility, self-care, usual activities, pain/discomfort, anxiety/depression and any of the five dimensions) by body mass index (BMI). With reference to underweight i.e. BMI <18Kg/m^2 (OR=1), individuals with overweight (BMI>25Kg/m^2) or obesity (BMI>30Kg/m^2) reported significantly greater problems in all five domains.

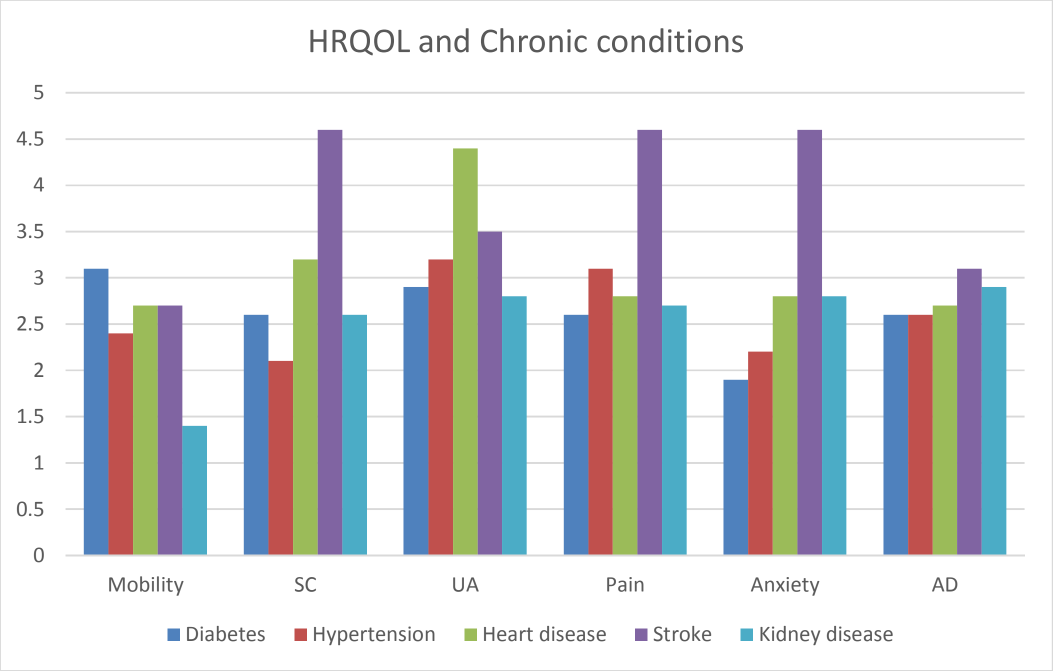

Figure 2.f. shows the odds of moderate or severe difficulties in EQ5D domains by chronic conditions. Compared to those without chronic conditions, individuals with self-reported diabetes, hypertension, heart disease, stroke and kidney disease had twice greater problems in all five domains.

For peer review only - http://bmjopen.bmj.com/site/about/guidelines.xhtml

Abbreviations: AD – Any Dimension, HRQOL – Health-related quality of life, SC – Self-Care, UA – Usual Activities,

For peer review only

For peer review only - http://bmjopen.bmj.com/site/about/guidelines.xhtml

Table 2 - Mean EQ-VAS and percentages of respondents reporting moderate or severe problems by various subgroups

	No. of respondents	EQ-VAS Mean	95%CI	Mobility (%)	95%CI	Self-care (%)	95%CI	Usual activities (%)	95%CI	Pain/discomfort (%)	95%CI	Anxiety/ depression (%)	95%CI
**Cities**													
Chennai	6906	70.79	[70.10,71.49]	17.3	[15.4,19.4]	7.8	[6.5,9.2]	7.7	[6.6,9.0]	10.3	[9.1,11.7]	11.4	[9.8,13.1]
Delhi	5364	78.88	[77.89,79.88]	14.1	[11.9,16.7]	1.6	[1.2,2.1]	4.4	[3.4,5.7]	19.4	[16.9,22.2]	5.2	[4.0,6.6]
Karachi	4017	73.23	[72.63,73.82]	10.4	[9.4,11.5]	1.9	[1.5,2.4]	5.6	[4.9,6.4]	12.9	[11.8,14.2]	7.2	[6.3,8.1]
**Employment status**													
Employed	7635	77.29	[76.45,78.14]	8.7	[7.7,9.9]	2.4	[1.8,3.1]	2.8	[2.3,3.4]	9.3	[8.3,10.4]	6.2	[5.4,7.1]
Student	361	77.5	[75.26,79.73]	6.5	[4.1,10.0]	1.5	[0.7,3.4]	2.6	[1.3,4.9]	8.3	[5.4,12.7]	7.0	[4.4,10.8]
Homemakers	6781	71.62	[70.75,72.49]	20.9	[18.9,22.9]	5.7	[4.8,6.8]	9.0	[7.8,10.4]	19.7	[17.6,22.0]	9.9	[8.9,11.0]
Retired	765	71.66	[69.98,73.34]	21.0	[17.7,24.6]	7.7	[5.7,10.3]	11.0	[8.5,14.0]	18.1	[15.0,21.8]	9.0	[7.0,11.5]
Unemployed	743	68.38	[66.87,69.90]	17.1	[13.6,21.2]	7.7	[5.7,10.5]	9.5	[7.3,12.4]	16.1	[13.2,19.6]	12.3	[9.8,15.2]
**Income class**													
Low income group (INR <10000 or US$ 155)	11537	73.44	[72.73,74.14]	15.2	[13.9,16.7]	4.8	[4.1,5.7]	6.8	[6.0,7.6]	14	[12.7,15.4]	9.2	[8.3,10.1]
Middle income group (INR 10000-20000 or US$ 155-310)	2667	75.87	[74.81,76.94]	14.6	[12.6,16.8]	3.5	[2.6,4.7]	4.9	[3.9,6.1]	14.5	[12.4,17.0]	6.4	[5.3,7.6]
High income group (INR >20000 or US$>310)	1975	77.17	[75.95,78.39]	11.0	[8.8,13.8]	1.5	[1.0,2.2]	3.4	[2.3,4.9]	15.5	[12.8,18.6]	5.0	[3.9,6.4]
**Education status**													
Up to primary school	3604	71.18	[70.11,72.24]	21.6	[19.5,24.0]	5.6	[4.7,6.7]	9.8	[8.5,11.2]	20.8	[18.5,23.3]	10.9	[9.6,12.4]
Secondary school	9924	74.34	[73.56,75.13]	14.0	[12.6,15.5]	4.5	[3.7,5.3]	5.8	[5.1,6.7]	13.0	[11.8,14.4]	8.1	[7.3,9.0]
Graduation and above	2759	77.94	[76.99,78.88]	8.3	[6.7,10.2]	1.6	[1.1,2.2]	2.3	[1.7,3.1]	10.8	[9.0,12.8]	5.0	[4.1,6.0]
**Marital Status**													
Single	1177	78.27	[76.94,79.60]	6.7	[5.2,8.7]	1.6	[0.9,2.9]	2.0	[1.2,3.2]	7.1	[5.5,9.2]	5.4	[3.9,7.5]
Married	14217	74.36	[73.67,75.05]	14.2	[13.0,15.6]	4.1	[3.5,4.8]	5.7	[5.0,6.5]	14	[12.6,15.4]	7.8	[7.1,8.6]

For peer review only

For peer review only - http://bmjopen.bmj.com/site/about/guidelines.xhtml

Widowed	838	67.47	[66.15,68.79]	34.5	[30.5,38.8]	10.2	[8.0,12.9]	18.7	[15.8,22.1]	32.7	[29.3,36.2]	18.2	[15.4,21.3]
Separated/Divorced	55	65.33	[57.91,72.75]	24.6	[11.8,44.3]	10.2	[4.6,21.3]	20.6	[8.8,41.0]	22.3	[12.2,37.2]	25.1	[14.9,39.2]
**BMI (Kg/m^2)**													
underweight (<18)	756	74.03	[72.30,75.77]	14.9	[11.7,18.8]	3.0	[1.7,5.3]	5.4	[3.7,7.9]	12.9	[10.1,16.5]	9.0	[6.3,12.7]
Normal (18-25)	5278	75.15	[74.35,75.94]	11.8	[10.4,13.3]	3.5	[2.8,4.2]	4.8	[4.0,5.7]	11.5	[10.2,13.0]	7.5	[6.6,8.7]
Overweight (25-30)	4190	73.62	[72.72,74.52]	15.2	[13.4,17.2]	4.8	[3.8,6.0]	6.2	[5.2,7.3]	14.4	[12.6,16.4]	8.2	[7.2,9.4]
Obesity (>30)	2249	70.4	[69.57,71.22]	22.3	[19.9,24.9]	6.4	[5.1,8.0]	9.1	[7.6,10.9]	20.7	[18.2,23.6]	10	[8.5,11.8]
**Tobacco use (Smoke/Chew/other forms)**													
Never user	12215	74.12	[73.44,74.80]	15.4	[14.0,16.8]	4.5	[3.8,5.3]	6.4	[5.6,7.2]	14.6	[13.1,16.2]	8.1	[7.3,9.0]
Current user	3758	75.33	[74.33,76.32]	11.8	[10.4,13.4]	3.1	[2.4,4.1]	4.6	[3.8,5.6]	12.9	[11.6,14.5]	7.9	[6.7,9.2]
Former user	314	70.15	[67.00,73.29]	17.6	[13.4,22.8]	4.7	[2.7,8.1]	8.3	[5.6,12.3]	18.1	[13.6,23.7]	12.4	[8.8,17.2]
**Chronic conditions (self-reported)**													
No	12498	76.21	[75.49,76.93]	11.9	[10.7,13.1]	3.5	[2.8,4.2]	4.5	[3.9,5.2]	11.2	[10.1,12.5]	6.8	[6.0,7.6]
Yes	4699	67.36	[66.62,68.09]	24.6	[22.4,27.0]	6.9	[5.9,7.9]	11.8	[10.5,13.2]	25.5	[23.3,27.9]	13.2	[12.0,14.5]
**Diabetes**													
No diabetes	4610	75.17	[74.41,75.92]	10.1	[8.9,11.4]	3.3	[2.6,4.1]	4.2	[3.5,5.0]	10.3	[8.8,12.0]	7.5	[6.5,8.6]
Pre-diabetes	5449	74.48	[73.59,75.37]	15.5	[13.6,17.5]	4.2	[3.3,5.2]	6.0	[5.0,7.2]	14.6	[12.9,16.4]	8.0	[7.0,9.1]
Newly diagnosed	2015	74.35	[73.39,75.30]	17.2	[14.7,20.1]	5.0	[3.8,6.5]	8.1	[6.5,10.0]	17.2	[15.0,19.6]	8.5	[7.1,10.1]
Self-reported Diabetes	1661	65.99	[64.86,67.12]	20.9	[18.9,23.1]	6.3	[5.2,7.5]	9.5	[8.3,11.0]	19.8	[18.1,21.7]	10.5	[9.3,11.8]
**Hypertension**													
Normotension	5695	74.82	[74.06,75.57]	12.8	[11.4,14.3]	3.8	[3.0,4.9]	4.7	[3.9,5.6]	11.6	[9.9,13.6]	6.9	[6.0,8.0]
Prehypertension	4717	76.02	[75.09,76.95]	12.7	[11.2,14.4]	3.7	[2.9,4.6]	5.1	[4.3,6.0]	12.3	[11.0,13.8]	6.5	[5.6,7.5]
Newly diagnosed	2780	75.85	[74.83,76.87]	12.9	[11.0,15.1]	3.0	[2.4,3.8]	5.1	[4.2,6.3]	12.6	[11.0,14.3]	8.4	[7.2,9.9]
Self-reported	2397	66.79	[65.88,67.71]	18.8	[16.8,21.0]	4.9	[4.2,5.7]	8.7	[7.6,9.9]	19.6	[17.8,21.4]	10.8	[9.7,12.0]
**Heart Disease**													
No	15842	74.61	[73.92,75.29]	14.2	[12.9,15.5]	4.0	[3.4,4.7]	5.6	[5.0,6.4]	13.8	[12.6,15.2]	7.9	[7.2,8.6]
Yes	445	63.33	[61.36,65.29]	31.2	[25.7,37.3]	11.7	[8.3,16.2]	20.8	[16.7,25.7]	31.4	[26.5,36.7]	19.0	[15.0,23.8]
**Stroke**													
No	16203	74.38	[73.69,75.07]	14.5	[13.3,15.8]	4.1	[3.5,4.8]	6.0	[5.3,6.7]	14.1	[12.9,15.5]	8.1	[7.4,8.8]
Yes	84	62.41	[58.58,66.25]	31.9	[22.0,43.7]	16.8	[10.0,26.7]	18.1	[11.1,28.1]	43.3	[32.3,55.1]	21.2	[13.2,32.2]

For peer review only

For peer review only - http://bmjopen.bmj.com/site/about/guidelines.xhtml

**Kidney disease**															
No	16175	74.41	[73.72,75.09]	14.6	[13.3,15.9]	4.1	[3.6,4.8]	6.0	[5.3,6.7]	14.1	[12.9,15.5]	8.1	[7.3,8.8]
Yes	112	62.57	[59.41,65.74]	19.2	[13.1,27.2]	10	[5.7,17.0]	15.1	[9.9,22.3]	31.4	[23.5,40.6]	20.7	[14.1,29.3]

NOTES: Tobacco use, Heart disease, Kidney disease were based on self-reports, newly diagnosed diabetes - defined as no self-reported diabetes and fasting blood glucose (FBG) of ≥126 mg/dl, or HbA1c ≥6.5%), pre-diabetes – no self-reported diabetes and FBG ≥100-125 mg/dl or HbA1c ≥5.7-6.4%), normoglycemia –no self-reported diabetes and FBG<100 mg/dl and HbA1c<5.7%, Newly diagnosed hypertension – defined as no self-reported hypertension and BP ≥140/90mmHg, prehypertension - no self-reported hypertension and BP: 120-139 / 80-89 mmHg and normotensive - no history of hypertension and BP <120/80 mmHg.

Abbreviations: INR: Indian rupees, mmHg – millimetre of mercury, mg/dl - Milligram/decilitre; US$ - United States Dollar

For peer review only

Table 3: Adjusted Odds ratio of reporting any problems in individuals with chronic conditions versus those without chronic conditions, by cities and gender

	Mobility		Self-care		Usual activities		Pain/discomfort		Anxiety/depression	
	**OR**	**95% CI**	**OR**	**95% CI**	**OR**	**95% CI**	**OR**	**95% CI**	**OR**	**95% CI**
**Overall**	1.6	[1.38,1.85]	1.4	[1.11,1.78]	1.8	[1.55,2.22]	2.1	[1.82,2.33]	1.7	[1.47,2.02]
Males	2.1	[1.56,2.56]	1.6	[1.11,2.48]	2.1	[1.45,3.14]	2.1	[1.74,2.55]	2.2	[1.63,2.94]
Females	1.4	[1.23,1.76]	1.3	[1.00,1.79]	1.8	[1.48,2.21]	2.1	[1.78,2.47]	1.5	[1.28,1.84]
**Chennai**	1.3	[1.07,1.72]	1.1	[0.82,1.48]	1.3	[1.09,1.74]	2.6	[1.79,3.96]	1.7	[1.38,2.08]
Males	1.6	[1.08,2.33]	1.1	[0.69,1.85]	1.2	[0.78,2.01]	1.9	[1.38,2.51]	1.9	[1.40,2.59]
Females	1.3	[0.95,1.72]	1.1	[0.75,1.58]	1.5	[1.13,1.93]	1.9	[1.49,2.56]	1.6	[1.20,2.09]
**Delhi**	2.1	[1.60,2.50]	1.9	[1.4, 2.5]	2.6	[1.79,3.96]	2.3	[1.68,3.09]	1.9	[1.32,2.89]
Males	2.5	[1.69,3.77]	5.4	[1.99,14.82]	4.1	[1.75,9.64]	2.2	[1.63,3.01]	3.6	[1.36,9.78]
Females	1.9	[1.44,2.46]	2.3	[1.29,4.16]	2.4	[1.54,3.62]	2.5	[1.84,3.35]	1.6	[1.13,2.39]
**Karachi**	1.5	[1.20,1.95]	1.8	[1.05,3.10]	2.3	[1.68,3.09]	1.9	[1.56,2.34]	1.7	[1.24,2.29]
Males	2.7	[1.56,4.86]	1.8	[0.74,4.36]	3.1	[1.66,5.97]	2.6	[1.60,4.14]	2.2	[1.30,3.73]
Females	1.3	[1.01,1.69]	2.2	[1.11,4.24]	2.1	[1.51,2.98]	1.7	[1.32,2.15]	1.4	[0.93,2.02]

NOTES: *Logistic regression model was adjusted for age, sex, income, education, marital status

Abbreviations: CI – confidence interval, OR – odds ratio

For peer review only

For peer review only - http://bmjopen.bmj.com/site/about/guidelines.xhtml

References

1. Rabin R, de Charro F. EQ-5D: a measure of health status from the EuroQol Group. Ann Med. 2001;33(5):337-43.
2. Todorich BM, Connor JR. Redox metals in Alzheimer's disease. Ann N Y Acad Sci. 2004;1012:171-8.
3. Kaplan RM, Anderson JP. A general health policy model: update and applications. Health Serv Res. 1988;23(2):203-35.
4. Coons SJ, Rao S, Keininger DL, Hays RD. A comparative review of generic quality-of-life instruments. Pharmacoeconomics. 2000;17(1):13-35.
5. Prabhakaran D, Jeemon P, Roy A. Cardiovascular Diseases in India: Current Epidemiology and Future Directions. Circulation. 2016;133(16):1605-20.
6. Fortin M, Lapointe L, Hudon C, Vanasse A, Ntetu AL, Maltais D. Multimorbidity and quality of life in primary care: a systematic review. Health Qual Life Outcomes. 2004;2:51.
7. Alonso J, Ferrer M, Gandek B, Ware JE, Jr., Aaronson NK, Mosconi P, et al. Health-related quality of life associated with chronic conditions in eight countries: results from the International Quality of Life Assessment (IQOLA) Project. Qual Life Res. 2004;13(2):283-98.
8. Schultz SE, Kopec JA. Impact of chronic conditions. Health Rep. 2003;14(4):41-53.
9. Patrick DL, Deyo RA. Generic and disease-specific measures in assessing health status and quality of life. Med Care. 1989;27(3 Suppl):S217-32.
10. Horsman J, Furlong W, Feeny D, Torrance G. The Health Utilities Index (HUI): concepts, measurement properties and applications. Health Qual Life Outcomes. 2003;1:54.
11. Furlong WJ, Feeny DH, Torrance GW, Barr RD. The Health Utilities Index (HUI) system for assessing health-related quality of life in clinical studies. Ann Med. 2001;33(5):375-84.
12. Brazier J, Roberts J, Deverill M. The estimation of a preference-based measure of health from the SF-36. J Health Econ. 2002;21(2):271-92.
13. McHorney CA. Health status assessment methods for adults: past accomplishments and future challenges. Annu Rev Public Health. 1999;20:309-35.
14. The World Health Organization Quality of Life Assessment (WHOQOL): development and general psychometric properties. Soc Sci Med. 1998;46(12):1569-85.
15. The World Health Organization Quality of Life assessment (WHOQOL): position paper from the World Health Organization. Soc Sci Med. 1995;41(10):1403-9.
16. Study protocol for the World Health Organization project to develop a Quality of Life assessment instrument (WHOQOL). Qual Life Res. 1993;2(2):153-9.
17. Centers for Disease Control and Prevention. Measuring healthy days: Population assessment of health-related quality of life. Centers for Disease Control and Prevention, Atlanta, Georgia 2000.
18. Gandek B, Sinclair SJ, Kosinski M, Ware JE, Jr. Psychometric evaluation of the SF-36 health survey in Medicare managed care. Health Care Financ Rev. 2004;25(4):5-25.
19. Nair M, Ali MK, Ajay VS, Shivashankar R, Mohan V, Pradeepa R, et al. CARRS Surveillance study: design and methods to assess burdens from multiple perspectives. BMC Public Health. 2012;12:701.
20. Pandit N, Dahiya R, Gupta R, Bali D, Kathuria A. Comparative evaluation of locally delivered minocycline and metronidazole in the treatment of periodontitis. Contemp Clin Dent. 2013;4(1):48-53.
21. Patel SA, Shivashankar R, Ali MK, Anjana RM, Deepa M, Kapoor D, et al. Is the "South Asian Phenotype" Unique to South Asians?: Comparing Cardiometabolic Risk Factors in the CARRS and NHANES Studies. Glob Heart. 2016;11(1):89-96 e3.
22. Deepa M, Grace M, Binukumar B, Pradeepa R, Roopa S, Khan HM, et al. High burden of prediabetes and diabetes in three large cities in South Asia: The Center for cArdio-metabolic Risk Reduction in South Asia (CARRS) Study. Diabetes Res Clin Pract. 2015;110(2):172-82.

For peer review only - http://bmjopen.bmj.com/site/about/guidelines.xhtml

23. Berg CJ, Ajay VS, Ali MK, Kondal D, Khan HM, Shivashankar R, et al. A cross-sectional study of the prevalence and correlates of tobacco use in Chennai, Delhi, and Karachi: data from the CARRS study. BMC Public Health. 2015;15:483.
24. Ali MK, Bhaskarapillai B, Shivashankar R, Mohan D, Fatmi ZA, Pradeepa R, et al. Socioeconomic status and cardiovascular risk in urban South Asia: The CARRS Study. Eur J Prev Cardiol. 2016;23(4):408-19.
25. Kind P, Dolan P, Gudex C, Williams A. Variations in population health status: results from a United Kingdom national questionnaire survey. BMJ. 1998;316(7133):736-41.
26. Sun H, Zhang Q, Luo X, Quan H, Zhang F, Liu C, et al. Changes of adult population health status in China from 2003 to 2008. PLoS One. 2011;6(12):e28411.
27. Sun S, Chen J, Johannesson M, Kind P, Xu L, Zhang Y, et al. Regional differences in health status in China: population health-related quality of life results from the National Health Services Survey 2008. Health Place. 2011;17(2):671-80.
28. Sun S, Chen J, Johannesson M, Kind P, Xu L, Zhang Y, et al. Population health status in China: EQ-5D results, by age, sex and socio-economic status, from the National Health Services Survey 2008. Qual Life Res. 2011;20(3):309-20.
29. Measuring Self-Reported Population Health: An International Perspective based on EQ-5D. In: Szende A, Williams, A., ed. On behalf of: The EuroQol Group's International Task Forceon Self-Reported Health; 2004.
30. Sayed BA, French MT. To your health!: Re-examining the health benefits of moderate alcohol use. Soc Sci Med. 2016;167:20-8.
31. Mauro PM, Canham SL, Martins SS, Spira AP. Substance-use coping and self-rated health among US middle-aged and older adults. Addict Behav. 2015;42:96-100.
32. Kindig DA BB, Remington PL. Mobilizing Action Toward Community Health (MATCH): metrics, incentives, and partnerships for population health. . Prev Chronic Dis. 2010;7(5).
33. Hennessy CH, Moriarty DG, Zack MM, Scherr PA, Brackbill R. Measuring health-related quality of life for public health surveillance. Public Health Rep. 1994;109(5):665-72.
34. Dominick KL, Ahern FM, Gold CH, Heller DA. Relationship of health-related quality of life to health care utilization and mortality among older adults. Aging Clin Exp Res. 2002;14(6):499-508.
35. Prazeres F, Santiago L. Relationship between health-related quality of life, perceived family support and unmet health needs in adult patients with multimorbidity attending primary care in Portugal: a multicentre cross-sectional study. Health Qual Life Outcomes. 2016;14(1):156.
36. Polat U, Bayrak Kahraman B, Kaynak I, Gorgulu U. Relationship among health-related quality of life, depression and awareness of home care services in elderly patients. Geriatr Gerontol Int. 2016;16(11):1211-9.
37. Lim JW, Paek MS. The relationship between communication and health-related quality of life in survivorship care for Chinese-American and Korean-American breast cancer survivors. Support Care Cancer. 2013;21(4):1157-66.
38. Badia X, Herdman M, Kind P. The influence of ill-health experience on the valuation of health. Pharmacoeconomics. 1998;13(6):687-96.
39. Sullivan PW, Ghushchyan V. Preference-Based EQ-5D index scores for chronic conditions in the United States. Med Decis Making. 2006;26(4):410-20.
40. Ko Y, Coons SJ. Self-reported chronic conditions and EQ-5D index scores in the US adult population. Curr Med Res Opin. 2006;22(10):2065-71.
41. Sakthong P, Sonsa-Ardjit N, Sukarnjanaset P, Munpan W. Psychometric properties of the EQ-5D-5L in Thai patients with chronic diseases. Qual Life Res. 2015;24(12):3015-22.
42. Tan Z, Liang Y, Liu S, Cao W, Tu H, Guo L, et al. Health-related quality of life as measured with EQ-5D among populations with and without specific chronic conditions: a population-based survey in Shaanxi Province, China. PLoS One. 2013;8(7):e65958.
43. McPherson K, Myers J, Taylor WJ, McNaughton HK, Weatherall M. Self-valuation and societal valuations of health state differ with disease severity in chronic and disabling conditions. Med Care. 2004;42(11):1143-51.

For peer review only - http://bmjopen.bmj.com/site/about/guidelines.xhtml

44. Lam JM, Wodchis WP. The relationship of 60 disease diagnoses and 15 conditions to preference-based health-related quality of life in Ontario hospital-based long-term care residents. Med Care. 2010;48(4):380-7.
45. Hutchinson AF, Graco M, Rasekaba TM, Parikh S, Berlowitz DJ, Lim WK. Relationship between health-related quality of life, comorbidities and acute health care utilisation, in adults with chronic conditions. Health Qual Life Outcomes. 2015;13:69.
46. Hevey D, McGee HM, Horgan J. Relationship of initial level of distress to changes in health-related quality of life during cardiac rehabilitation or usual care. Psychosom Med. 2007;69(8):793-7.
47. Arnlind MH, Nokela M, Rehnberg C, Jonsson EW. The relationship between pharmaceutical costs, disease severity, and health-related quality of life in asthmatics in Swedish primary care. J Asthma. 2006;43(8):585-91.
48. Mo F, Choi BC, Li FC, Merrick J. Using Health Utility Index (HUI) for measuring the impact on health-related quality of Life (HRQL) among individuals with chronic diseases. ScientificWorldJournal. 2004;4:746-57.
49. Lubetkin EI, Jia H, Franks P, Gold MR. Relationship among sociodemographic factors, clinical conditions, and health-related quality of life: examining the EQ-5D in the U.S. general population. Qual Life Res. 2005;14(10):2187-96.
50. Jia H, Lubetkin EI. The impact of obesity on health-related quality-of-life in the general adult US population. J Public Health (Oxf). 2005;27(2):156-64.
51. Chen J, Wong, C.K.H., McGhee,S.M., Pang,P.K.P., Yu, W.C. A Comparison between the EQ-5D and the SF-6D in Patients with Chronic Obstructive Pulmonary Disease (COPD). PLoS One. 2014;9(11).
52. Salaffi F, Carotti M, Ciapetti A, Gasparini S, Grassi W. A comparison of utility measurement using EQ-5D and SF-6D preference-based generic instruments in patients with rheumatoid arthritis. Clin Exp Rheumatol. 2011;29(4):661-71.
53. Wu J, Han Y, Zhao FL, Zhou J, Chen Z, Sun H. Validation and comparison of EuroQoL-5 dimension (EQ-5D) and Short Form-6 dimension (SF-6D) among stable angina patients. Health Qual Life Outcomes. 2014;12:156.
54. Petrou S, Hockley C. An investigation into the empirical validity of the EQ-5D and SF-6D based on hypothetical preferences in a general population. Health Econ. 2005;14(11):1169-89.
55. Obradovic M, Lal A, Liedgens H. Validity and responsiveness of EuroQol-5 dimension (EQ-5D) versus Short Form-6 dimension (SF-6D) questionnaire in chronic pain. Health Qual Life Outcomes. 2013;11:110.
56. Kuspinar A, Mayo NE. A review of the psychometric properties of generic utility measures in multiple sclerosis. Pharmacoeconomics. 2014;32(8):759-73.
57. Wu SC, Li CY, Ke DS. The agreement between self-reporting and clinical diagnosis for selected medical conditions among the elderly in Taiwan. Public Health. 2000;114(2):137-42.
58. Molenaar EA, Van Ameijden EJ, Grobbee DE, Numans ME. Comparison of routine care self-reported and biometrical data on hypertension and diabetes: results of the Utrecht Health Project. Eur J Public Health. 2007;17(2):199-205.
59. Robinson JR, Young TK, Roos LL, Gelskey DE. Estimating the burden of disease. Comparing administrative data and self-reports. Med Care. 1997;35(9):932-47.
60. Okura Y, Urban LH, Mahoney DW, Jacobsen SJ, Rodeheffer RJ. Agreement between self-report questionnaires and medical record data was substantial for diabetes, hypertension, myocardial infarction and stroke but not for heart failure. J Clin Epidemiol. 2004;57(10):1096-103.

For peer review only - http://bmjopen.bmj.com/site/about/guidelines.xhtml

Web-appendix (supplementary file)

Appendix 1: The distribution of the respondents EQ-VAS values

EQ-VAS score	Number	Percentage (%)	Cum. Percentage (%)
10-	29	0.18	0.18
20-	72	0.44	0.62
30-	88	0.54	1.16
40-	365	2.24	3.4
50-	2,056	12.62	16.03
60-	1,979	12.15	28.18
70-	3,728	22.89	51.07
80-	4,026	24.72	75.78
90-	2,594	15.93	91.71
100-	1,350	8.29	100

Notes: EQ-VAS: European Quality of Life – Visual Analogue Scale, Cum. Percentage – Cumulative percentage

For peer review only

Appendix 2: Relationship between EQ-VAS and EQ5D across major sub-groups

Dependent variable	Mobility	Self-care	Usual care	Pain / discomfort	Anxiety / Depression	Observation
Overall	-10.5 (0.6)	-9.9 (0.9)	-12.4 (0.7)	-10.9 (0.6)	-8.9 (0.8)	16287
Gender						
**Male**	-12.0 (0.6)	-11.4 (1.1)	-14.6 (0.9)	-11.9 (0.6)	-12.1 (0.7)	7760
**Female**	-8.1 (0.4)	-7.9 (0.7)	-9.9 (0.6)	-7.7 (0.4)	-9.1 (0.5)	8527
Age groups						
**Young (20-44 yrs)**	-9.9 (0.5)	-11.0 (0.9)	-12.0 (0.8)	-8.8 (0.5)	-9.3 (0.6)	9603
**Middle (45-60 yrs)**	-9.0 (0.5)	-10.8 (0.9)	-11.3 (0.8)	-9.0 (0.5)	-10.7 (0.6)	5544
**Elderly (>60 yrs)**	-11.1 (1.0)	-11.7 (1.6)	-13.1 (1.3)	-11.0 (1.1)	-13.2 (1.4)	1140
Income						
**Low income group (INR <10000 or US$ 155)**	-10.6 (0.4)	-12.1 (0.7)	-13.2 (0.6)	-10.2 (0.4)	-10.7 (0.5)	11537
**Middle income group (INR 10000-20000 or US$ 155-310)**	-10.2 (0.8)	-8.9 (1.6)	-10.7 (1.3)	-9.2 (0.8)	-11.8 (1.1)	2667
**High income group (INR >20000 or US$>310)**	-12 (1.0)	-14.4(2.5)	-15.6 (1.7)	-10.5 (0.9)	-8.2 (1.6)	1975
Level of education						
**Up to primary school**	-10.8 (0.6)	-12.8 (1.1)	-12.6 (0.8)	-9.9 (0.6)	-11.6 (0.8)	3604
**Secondary school**	-9.9 (0.4)	-11.1 (0.8)	-12.2 (0.7)	-9.3 (0.4)	-10.2 (0.5)	9924
**Graduation and above**	-8.9 (1.0)	-8.3 (2.1)	-11.4 (1.8)	-9.8 (0.9)	-8.3 (1.3)	2759
Marital status						
**Single**	-8.4 (1.9)	-9.4 (3.8)	-15.2 (3.3)	-9.0 (1.8)	-10.8 (2.0)	1177
**Married**	-10.3 (0.4)	-11.5 (0.6)	-12.3 (0.5)	-9.5 (0.4)	-10.3 (0.5)	14217
**Widowed**	-9.9 (1.2)	-11.1 (1.8)	-12.9 (1.4)	-10.1 (1.2)	-11.8 (1.5)	838
**Divorce**	-18.6 (5.8)	-14.1 (7.0)	-24.2 (6.0)	-15.1 (5.6)	-15.6 (5.1)	55
Tobacco use						
**No**	-10.1 (0.4)	-11.4 (0.6)	-12.4 (0.5)	-9.8 (0.4)	-10.5 (0.5)	12529
**Yes**	-13.4 (0.8)	-15.5 (1.5)	-16.5 (1.2)	-11.1 (0.7)	-12.6 (0.9)	3758
Alcohol use						
**No**	-10.6 (0.4)	-12.1 (0.6)	-13.1 (0.5)	-10.2 (0.4)	-11.0 (0.5)	13911
**Yes**	-10.6 (1.1)	-10.7 (2.0)	-11.4 (1.9)	-8.2 (1.0)	-10.6 (1.2)	2376
BMI						
**Underweight**	-10.6 (2.4)	-15.6 (5.1)	-15.4 (3.9)	-10.5 (2.9)	-7.8 (4.8)	756
**Normal weight**	-10.6 (0.9)	-11.6(1.5)	-13.6 (1.2)	-9.2 (0.9)	-9.7 (1.2)	5278
**Overweight**	-10.3 (1.1)	-12.1 (1.2)	-11.6 (1.3)	-7.7 (0.9))	-8.9 (1.2)	4190
**Obesity**	-7.4 (0.9)	-9.9 (1.7)	-11.4 (1.3)	-8.0 (0.9)	-8.6 (1.4)	2249
Diabetes						
**Diabetes (diagnosed or self-reported)**	-10.0 (0.6)	-11.6 (1.1)	-11.6 (0.9)	-9.7 (0.6)	-11.4 (0.8)	3676
**Pre diabetes**	-10.2 (0.6)	-13.5 (1.0)	-14.1 (0.8)	-9.4 (0.6)	-10.6 (0.7)	5449
**No diabetes**	-10.9 (0.7)	-11.0 (1.2)	-12.6 (1.1)	-9.9 (0.7)	-10.0 (0.8)	4610
Hypertension						

For peer review only - http://bmjopen.bmj.com/site/about/guidelines.xhtml

**Diagnosed or Self-reported hypertension**	-11.2 (0.6)	-14.7 (1.0)	-14.1 (0.8)	-10.8 (0.6)	-10.8 (0.7)	5074
**Pre hypertension**	-10.9 (0.7)	-12.4 (1.2)	-14.1 (1.0)	-9.7 (0.7)	-11.2 (0.9)	4717
**No hypertension**	-9.3 (0.6)	-9.6 (1.0)	-10.4 (0.9)	-8.4 (0.6)	-10.0 (0.7)	5695
Heart disease						
**No**	-10.5 (0.3)	-11.9 (0.6)	-13.0 (0.5)	-9.9 (0.3)	-10.7 (0.4)	15842
**Yes**	-9.0 (1.6)	-9.0 (2.5)	-9.5 (1.9)	-7.4 (1.7)	-11.8 (2.0)	445
Stroke						
**No**	-10.7 (0.3)	-11.9 (0.6)	-13.1 (0.5)	-10.0 (0.3)	-10.8 (0.4)	16203
**Yes**	-13.7 (3.6)	-18.4 (4.4)	-19.2 (4.1)	-12.2 (3.5)	-16.3 (4.1)	84
Kidney disease						
**No**	-10.7 (0.3)	-11.9 (0.6)	-13.1 (0.5)	-9.9 (0.3)	-10.8 (0.4)	16175
**Yes**	-11.6 (3.7)	-21.2 (4.8)	-14.3 (4.2)	-16.4 (3.2)	-16.3 (3.7)	112

NOTES: Tobacco use, Heart disease, Kidney disease were based on self-reports, newly diagnosed diabetes - defined as no self-reported diabetes and fasting blood glucose (FBG) of ≥126 mg/dl, or HbA1c ≥6.5%), pre-diabetes – no self-reported diabetes and FBG ≥100-125 mg/dl or HbA1c ≥5.7-6.4%), normoglycemia – no self-reported diabetes and FBG<100 mg/dl and HbA1c<5.7%, Newly diagnosed hypertension – defined as no self-reported hypertension and BP ≥140/90mmHg, prehypertension - no self-reported hypertension and BP: 120-139 / 80-89 mmHg and normotensive - no history of hypertension and BP <120/80 mmHg. INR: Indian rupees, mmHg – millimeter of mercury, mg/dl - Milligram/deciliter; yrs – years; US$ - United States Dollar

For peer review only

Appendix 3 - The comparison of HRQOL as measured by EQ5D-VAS among different countries

**Countries, year of study**	**N**	**EQ-VAS**	**Percentages with any difficulties in EQ5D domain**					
		Mean	Mobility	Self-care	Usual activities	Pain / Discomfort	Anxiety / Depression	Any dimension
India, **Delhi (age≥20), 2011**	5,365	78.9	14.1	1.6	4.4	8.0	9.5	27.8
India, **Chennai, (age≥20), 2011**	6,903	70.8	17.3	7.8	7.7	9.0	8.9	29.7
Pakistan **(Karachi), (age≥20), 2011**	4,016	73.2	10.4	1.9	5.6	8.7	9.3	17.9
China **(age≥18), 2008**	2,991	77.0	4.9	2.0	3.3	18.0	6.1	22.4
UK **(age≥18), 1998**	3395	82.5	18.4	4.2	16.3	33.0	20.9	43.1
USA **(age≥18) , 1998**	427	82.2	14.0	3.0	14.0	40.0	24.0	na
Japan **(age≥20) , 1998**	620	77.8	7.2	1.8	5.2	20.0	8.5	25.0
Spain **(age≥15) , 1998**	12,245	71.1	11.2	2.0	6.9	26.3	12.5	33.0
Canada **(age≥18), 1997**	1518	78.7	22.2	4.0	19.1	43.6	28.6	53.0
Sweden **(age≥18), 1998**	3069	83.5	10.0	2.0	8.0	42.0	30.0	na
Finland **(age≥18), 1992**	2411	79.4	20.0	5.0	18.0	39.0	14.0	na
Germany **(age≥18), 1998**	337	82.2	18.0	3.0	13.0	37.0	18.0	na
Belgium **(age≥18), 2001**	1274	81.0	13.0	3.0	15.0	42.0	21.0	na
New Zealand **(age≥18), 1999**	1328	81.3	17.0	4.0	18.0	37.0	20.0	na
Zimbabwe **(age≥18), 2000**	2350	76.1	20.0	7.0	18.0	41.0	40.0	na
Armenia **(age≥18), 2002**	2222	66.6	26.0	13.0	28.0	64.0	52.0	na

NOTES: EQ5D-VAS: European Quality of Life 5 Dimension – Visual Analogue Scale; na – not available

For peer review only - http://bmjopen.bmj.com/site/about/guidelines.xhtml

STROBE 2007 (v4) Statement—Checklist of items that should be included in reports of *cross-sectional studies*

Section/Topic	Item #	Recommendation	Reported on page #
**Title and abstract**	1	(*a*) Indicate the study's design with a commonly used term in the title or the abstract	1, 6
		(*b*) Provide in the abstract an informative and balanced summary of what was done and what was found	6
**Introduction**			
Background/rationale	2	Explain the scientific background and rationale for the investigation being reported	8,9
Objectives	3	State specific objectives, including any prespecified hypotheses	9
**Methods**			
Study design	4	Present key elements of study design early in the paper	9
Setting	5	Describe the setting, locations, and relevant dates, including periods of recruitment, exposure, follow-up, and data collection	9
Participants	6	(*a*) Give the eligibility criteria, and the sources and methods of selection of participants	9, 10
Variables	7	Clearly define all outcomes, exposures, predictors, potential confounders, and effect modifiers. Give diagnostic criteria, if applicable	10
Data sources/ measurement	8*	For each variable of interest, give sources of data and details of methods of assessment (measurement). Describe comparability of assessment methods if there is more than one group	10, 11
Bias	9	Describe any efforts to address potential sources of bias	*12 (adjusted regression model was run to adjust for potential confounders)*
Study size	10	Explain how the study size was arrived at	Not applicable – secondary data analysis
Quantitative variables	11	Explain how quantitative variables were handled in the analyses. If applicable, describe which groupings were chosen and why	12

For peer review only

Statistical methods	12	(*a*) Describe all statistical methods, including those used to control for confounding	12
		(*b*) Describe any methods used to examine subgroups and interactions	Not applicable
		(*c*) Explain how missing data were addressed	Not applicable
		(*d*) If applicable, describe analytical methods taking account of sampling strategy	Not applicable
		(*e*) Describe any sensitivity analyses	Not applicable
**Results**			
Participants	13*	(a) Report numbers of individuals at each stage of study—eg numbers potentially eligible, examined for eligibility, confirmed eligible, included in the study, completing follow-up, and analysed	13
		(b) Give reasons for non-participation at each stage	Not applicable
		(c) Consider use of a flow diagram	Not applicable
Descriptive data	14*	(a) Give characteristics of study participants (eg demographic, clinical, social) and information on exposures and potential confounders	13
		(b) Indicate number of participants with missing data for each variable of interest	13
Outcome data	15*	Report numbers of outcome events or summary measures	13
Main results	16	(*a*) Give unadjusted estimates and, if applicable, confounder-adjusted estimates and their precision (eg, 95% confidence interval). Make clear which confounders were adjusted for and why they were included	13
		(*b*) Report category boundaries when continuous variables were categorized	13
		(*c*) If relevant, consider translating estimates of relative risk into absolute risk for a meaningful time period	Not applicable
Other analyses	17	Report other analyses done—eg analyses of subgroups and interactions, and sensitivity analyses	13 - 15
**Discussion**			
Key results	18	Summarise key results with reference to study objectives	15
Limitations	19	Discuss limitations of the study, taking into account sources of potential bias or imprecision. Discuss both direction and magnitude of any potential bias	19
Interpretation	20	Give a cautious overall interpretation of results considering objectives, limitations, multiplicity of analyses, results from similar studies, and other relevant evidence	16-18
Generalisability	21	Discuss the generalisability (external validity) of the study results	18 - 19
**Other information**			
Funding	22	Give the source of funding and the role of the funders for the present study and, if applicable, for the original study on which the present article is based	20

For peer review only

*Give information separately for cases and controls in case-control studies and, if applicable, for exposed and unexposed groups in cohort and cross-sectional studies.

Note: An Explanation and Elaboration article discusses each checklist item and gives methodological background and published examples of transparent reporting. The STROBE checklist is best used in conjunction with this article (freely available on the Web sites of PLoS Medicine at http://www.plosmedicine.org/, Annals of Internal Medicine at http://www.annals.org/, and Epidemiology at http://www.epidem.com/). Information on the STROBE Initiative is available at www.strobe-statement.org.

For peer review only

BMJ Open

Health-related Quality of Life Variations by Socio-demographic Factors and Chronic Conditions in three metropolitan cities of South Asia: The CARRS Study

Journal:	*BMJ Open*
Manuscript ID	bmjopen-2017-018424.R1
Article Type:	Research
Date Submitted by the Author:	07-Sep-2017
Complete List of Authors:	Singh, Kavita; All India Institute of Medical Sciences, Endocrinology & Metabolism; Public Health Foundation of India<br>Kondal, Dimple; Public Health Foundation of India<br>Shivashankar, Roopa; Public Health Foundation of India,<br>Ali, Mohammed; Emory University, Hubert Department of Global Health<br>Pradeepa, Rajendra; Madras Diabetes Research Foundation & Dr.Mohan's Diabetes Specialities Centre, Epidemiology & Diabetology<br>Vamadevan, Ajay; Centre for Chronic Disease Control<br>Mohan, V; Madras Diabetes Research Foundation<br>Kadir, Muhammad ; Aga Khan University, Karachi, Pakistan, Community Health Sciences<br>Sullivan, Mark; University of Washington School of Medicine,<br>Tandon, Nikhil; All India Institute of Medical Sciences,<br>Narayan, K; Emory University School of Public Health, Global Health<br>Prabhakaran, Dorairaj; Centre for Chronic Disease Control, ; Public Health Foundation of India,
**Primary Subject Heading**:	Public health
Secondary Subject Heading:	Epidemiology, Global health, Health policy
Keywords:	Health related Quality of Life (HRQOL), EQ5D, Chronic conditions, South Asia, Visual Analogue Scale

SCHOLARONE™
Manuscripts

Title

Health-related Quality of Life Variations by Socio-demographic Factors and Chronic Conditions in three metropolitan cities of South Asia: The CARRS Study

For peer review only

For peer review only - http://bmjopen.bmj.com/site/about/guidelines.xhtml

Authors name, title, affiliation, address, contact details

Ms. Kavita Singh, MSc, PhD Candidate

Research Scientist, Centre for Chronic Conditions and Injuries (CCCI), Public Health Foundation of India (PHFI), and

Centre for Chronic Disease Control (CCDC), and

All India Institute of Medical Sciences, New Delhi

4th floor, Plot no. 47, Sector-44, Gurgaon - 122 002, Haryana

Office Landline - +91-124 – 4781400

Fax – +91-124-4722901, Mobile – +91-9899691150

Email: kavita@ccdcindia.org; kavita.singh@phfi.org

Dr. Dimple Kondal, MSc, PhD

Biostatistician, Centre for Chronic Conditions and Injuries,

Public Health Foundation of India

4th floor, Plot no. 47, Sector-44, Gurgaon - 122 002, Haryana

Office Landline - +91-124-4781400

Fax – +91-124-4722901, Mobile – +91-9899691150

Email: dimple.kondal@phfi.org

Dr. Roopa Shivashankar, MD, MSc

Senior Research Scientist and Associate Professor,

Centre for Chronic Conditions and Injuries,

Public Health Foundation of India, and

Centre for Chronic Disease Control

4th floor, Plot no. 47, Sector-44, Gurgaon - 122 002, Haryana

For peer review only

For peer review only - http://bmjopen.bmj.com/site/about/guidelines.xhtml

Office Landline - +91-0124 – 4781400

Fax – +91-0124-4722901, Mobile – +91-9899691150

Email: roopa@ccdcindia.org; roopa.shivashankar@phfi.org

Dr. Mohammed K. Ali, MBChB, MSc, MBA

Associate Professor, Hubert Department of Global

Health and Department of Epidemiology,

Rollins School of Public Health, Emory University, and

1518 Clifton Road NE, Room 7041, Atlanta, GA, 30322, USA

Phone number: +1 404 727 9776

Email: mkali@emory.edu

Dr. Rajendra Pradeepa, PhD

Head, Research Operations, Madras Diabetes Research Foundation

No.4, Conran Smith Road, Gopalapuram, Chennai 600086, India

Phone number: +91-44-43968888

Email: guhapradeepa@gmail.com

Dr. Vamadevan S Ajay, MPH, PhD

Senior Research Scientist, Centre for Chronic Conditions and Injuries, Public Health

Foundation of India, and

Centre for Chronic Disease Control

4th Floor, Plot No. 47, Sector 44, Institutional Area

Gurgaon - 122 002, Haryana

Office Landline - +91-124-4781400

Email: ajay@ccdcindia.org

For peer review only - http://bmjopen.bmj.com/site/about/guidelines.xhtml

Dr. Viswanathan Mohan, MD, PhD

President and Director, Madras Diabetes Research Foundation

No.4, Conran Smith Road, Gopalapuram, Chennai 600086, India

Phone number: +91-44-43968888

Email: drmohans@diabetes.ind.in

Dr. Muhammad M. Kadir, MBBS, FCPS

Professor, Aga Khan University, Department of Community Health Sciences,

Stadium Road, Karachi 74800, Pakistan

Phone number: +92 21 34930051

Email: masood.kadir@aku.edu

Dr. Mark Daniel Sullivan, MD, PhD

Professor, Psychiatry and Behavioral Sciences

Adjunct Professor, Anesthesiology and Pain Medicine, Bioethics and Humanities

Medical Director, UW Telepain

University of Washington, Box 356560, Seattle, WA 98195

Phone: +1-(206)685-3184, Fax: +1-(206) 221-5414

Email: sullimar@uw.edu

Dr. Nikhil Tandon, MD, PhD

Professor and Head, Department of Endocrinology & Metabolism, All India Institute of Medical Sciences,

Biotechnology Block, 3rd Floor, Rm #312, Ansari Nagar, New Delhi - 110 029, India

Phone: +91-9818211663

For peer review only

For peer review only - http://bmjopen.bmj.com/site/about/guidelines.xhtml

Email: nikhil_tandon@hotmail.com

Dr. KM Venkat Narayan, MD.

Ruth and O. C. Hubert, Professor of Global Health and Epidemiology, Rollins School of Public Health,

Emory University

1518 Clifton Road NE, Room 7041, Atlanta, GA, 30322, USA

Phone number: +1 404 727 9776

Email: KNARAYA@emory.edu

Dr. Dorairaj Prabhakaran*, MD, DM.

Vice President (Research and Policy),

Public Health Foundation of India, and

Director, Centre for Chronic Disease Control,

Professor, London School of Hygiene and Tropical Medicine, UK

4th floor, Plot no. 47, Sector-44, Gurgaon - 122 002, Haryana

Office Landline - 0124 – 4781400

Email: dprabhakaran@ccdcindia.org

*Corresponding author

Word count:

Abstract: 237 words

Main article: 3,553

Number of figures: 2

Number of Tables: 3

Appendix: Tables: 3

For peer review only - http://bmjopen.bmj.com/site/about/guidelines.xhtml

For peer review only

For peer review only - http://bmjopen.bmj.com/site/about/guidelines.xhtml

ABSTRACT [237 words]

Objectives: Health-related quality of life (HRQOL) is a key indicator of health. However, HRQOL data from representative populations in South Asia are lacking. This study aims to describe HRQOL overall, by age, gender, and socio-economic status (SES), and examine the associations between selected chronic conditions and HRQOL in adults from three urban cities in South Asia.

Methods: We used data from 16287 adults aged ≥20 years from the baseline survey of the Centre for Cardiometabolic Risk Reduction in South Asia (CARRS) cohort (2010-11). HRQOL was measured using the European Quality of Life 5 dimension - Visual Analogue Scale (EQ5D-VAS), which measures health status on a scale of 0 (worst health status) – 100 (best possible health status).

Results: 16,284 participants completed EQ5D-VAS. Mean age was 42.4 (+/-13.3) years and 52.4% were women. 14% of the respondents reported problems in mobility, and pain/discomfort domains. Mean VAS score was 74 (95% confidence interval [CI]: 73.7, 74.2). Significantly lower health status was found in elderly (64.1), women (71.6), unemployed (68.4), less educated (71.2) and low-income group (73.4). Individuals with chronic conditions reported worse health status than those without (67.4 vs. 76.2): Prevalence Ratio: 1.8 [95%CI: 1.61, 2.04].

Conclusions: Our data demonstrate significantly lower HRQOL in key demographic groups and those with chronic conditions, which are consistent with previous studies. These data provide insights on inequalities in population health status, and potentially reveal unmet needs in the community to guide health policies.

Key words (5)

Health related Quality of Life (HRQOL), EQ5D, Chronic conditions, South Asia, Visual Analogue Scale

For peer review only

For peer review only - http://bmjopen.bmj.com/site/about/guidelines.xhtml

Strengths and limitations of this study

- This is the first population level health related quality of life (HRQOL) data from South Asia using EQ5D-VAS including three large metropolitan cities in India and Pakistan with a large sample size (16,284 adults aged ≥20 years).
- Our data provide the first baseline values to be used for monitoring population health status and analysed the relationships between selected chronic conditions and HRQOL.
- HRQOL data presented in this article could be used to complement national health targets by providing a measure of chronic disease burden based on perceived health status rather than solely on mortality and disease prevalence.
- Due to the cross-sectional nature of the data, the causal relationship between socio-economic parameters or chronic conditions and HRQOL cannot be determined.
- Many chronic conditions (respiratory, locomotor, cancer, etc.) were not included in the survey. Therefore, the ranking of most severe health conditions and associated HRQOL is not complete.

For peer review only

For peer review only - http://bmjopen.bmj.com/site/about/guidelines.xhtml

Main article [3553 words]

Introduction

Health-related Quality of Life (HRQOL) is a multidimensional concept that provides a broader perspective of health through conveying an individual's ability to function in physical, mental, and social domains of life (1). HRQOL is thus an essential patient-centred outcome measure, which is useful to guide health policies (2, 3). HRQOL is preferred over other health indicators (life expectancy, mortality, morbidity) for measuring chronic disease burden as it incorporates both length and quality of life (4). In South Asia, chronic conditions (diabetes, hypertension and heart diseases) occur at an early age (5) with detrimental effects on length and quality of life (6, 7). In addition, episodes and fear of hypoglycaemia, recurrent heart attacks, stroke and other long-term complications (kidney diseases, diabetic retinopathy) are not always measured as such, though they have a substantial adverse impact on an individual's overall health status (8). Therefore, it is important to quantify the effect of chronic conditions on individuals HRQOL.

There are several disease-specific (Chronic respiratory distress questionnaire, Arthritis Impact Measurement Scale) and generic instruments (Short Form 36, World Health Organization (WHO) – Quality of Life questionnaire, and European Quality of Life Five Dimensions – Visual Analogue Scale (EQ5D-VAS)) available to measure population HRQOL (4, 9-18). However, the EQ5D-VAS is favoured because it is generic, not specific to a particular disease, and it not only includes multidimensional measures of health profile in five dimensions (mobility, self-care, usual activities, pain/discomfort and anxiety/depression), but also includes the single-dimensional measure VAS, to summarize overall health status (1). Also, EQ5D-VAS has been applied and validated for

For peer review only - http://bmjopen.bmj.com/site/about/guidelines.xhtml

its use in many population surveys across the world, therefore, it makes the comparison of health status across populations easier.

Data on population HRQOL across socioeconomic status (SES) from South Asia are scarce, and little is known about the relative associations between different chronic conditions and individual HRQOL. The Centre for Cardio-metabolic Risk Reduction in South Asia (CARRS) study (19) had collected data on both EQ5D-VAS and selected chronic conditions from a large representative population of adults in urban South Asia. We used this opportunity to examine population HRQOL in this region. In this paper, we describe the variations in HRQOL by age, gender, and SES, and explore the relationships between selected chronic conditions and HRQOL in a representative sample of adults aged ≥20 years from three metropolitan cities in India and Pakistan. We also analysed the relationship between multidimensional EQ5D measures and single dimensional VAS across major subgroups.

Methods

Study design and setting

We obtained data from the baseline cross-sectional survey of the CARRS cohort (2010-11), which recruited a representative sample of non-pregnant adults aged ≥20 years from three urban cities: Chennai, Delhi, and Karachi. These metropolitan cities with large and heterogeneous populations in terms of demographic profile and economic transitions offer unique opportunities to assess variations in health status across different socio-economic groups. The detailed CARRS study design has been published elsewhere (19). Briefly, a multi-stage cluster random sampling strategy was used with wards (in Delhi and Chennai) or clusters (in Karachi) as the primary sampling units. Using the WHO STEPS survey “Kish method”, two participants, one male and one female,

For peer review only - http://bmjopen.bmj.com/site/about/guidelines.xhtml

For peer review only

aged ≥20 years (non-pregnant) and meeting the study eligibility criteria, were selected from each randomly selected household (19).

Study measures

Comprehensive and uniform data collection instruments were used to capture measurements in all three sites. A summary of all surveillance measures, methods, and instruments used in the study has been published in detail (19). Briefly, a questionnaire was administered to collect information regarding demographic, socio-economic, behavioural, and past and present health status of the participant.

Trained study staff measured anthropometric parameters (height, weight) using standardized techniques and blood pressure (BP) twice at each participant's home or at a medical camp organized in the community, after five minutes in a seated position using an electronic BP measuring device (Omron Dailan Co., China). If the difference between the first two systolic or diastolic BP readings was more than 10 mmHg or 5 mmHg, respectively, a third reading was taken. Average BP readings of the two/three readings were recorded in the study database. Additionally, fasting blood glucose and glycated haemoglobin (HbA1c) were measured. The overall response rates were 94.7% for questionnaire completion and 84.3% for blood tests.

Population health status was measured using the EQ5D-VAS questionnaire, which consisted of two components; health state description and self-rated health status on VAS. Health state description (profile) includes five dimensions (5D); mobility (walking ability), self-care (ability to wash or dress by oneself), usual activities (ability to work, study, housework), pain/discomfort, and anxiety/depression. The respondents self-rate their level of severity for each dimension using three levels (EQ5D-3L): having no problems, having some or moderate problems, or being unable to do/having extreme problems. The respondents were asked to choose one of the statements which best described their health status on the surveyed day. For example, three levels of 'mobility'

For peer review only - http://bmjopen.bmj.com/site/about/guidelines.xhtml

dimension were phrased as "I have no problems in walking," "I have some problems in walking," and "I am confined to bed". Given the possible permutations of different domains and response types, there are potentially 243 (=3^5) different health profiles. For overall health status, the respondents evaluated their health status using the VAS. The VAS asks respondents to mark health status on the day of the interview on a scale of 0 (worst health status) – 100 (best imaginable health status).

Covariates: Self-reported age at baseline in completed years was used and categorized into 20-24, 25-34, 35-44, 45-54, 55-64, 65-74 and ≥75. Based on participant responses, we categorized employment status into employed, student, housewife, retired, and unemployed. Income class was grouped into three categories based on household monthly income: low-income: less than 10,000 Indian rupees (INR) (equivalent to US$200), middle-income: 10,000-20,000 INR (US$200-400) and high-income strata: greater than 20,000 INR (US$400). We categorized highest education level attained into three categories - up to primary, secondary schooling, and graduates. The marital status was classified as: single, married, widowed, and divorced. Body mass index (Kg/m^2) international classification: of ≤17.9 was used to define the underweight, and 18.0-24.9=normal weight, 25.0-29.9=overweight and ≥30.0=obese. Lifestyle habits like tobacco use was classified based on self-reports as never, former and current user. Data on chronic conditions consisted of self-reported hypertension, diabetes, heart disease, stroke, and kidney disease. In addition, diabetes was categorized into self-reported, newly diagnosed (defined by no self-reported diabetes and fasting blood glucose (FBG) of ≥126 mg/dl, or HbA1c ≥6.5%), pre-diabetes (no self-reported diabetes and FBG ≥100-125 mg/dl or HbA1c ≥5.7-6.4%) and normoglycemia (no self-reported diabetes and FBG<100 mg/dl and HbA1c<5.7%). Similarly, we classified hypertension as: self-reported, newly diagnosed (no self-reported hypertension and BP ≥140/90mmHg),

For peer review only - http://bmjopen.bmj.com/site/about/guidelines.xhtml

prehypertension (no self-reported hypertension and BP: 120-139 / 80-89 mmHg) and normotensive (no history of hypertension and BP <120/80 mmHg).

Ethical considerations

The CARRS study has obtained institutional ethics approval from each of the participating institutions: Public Health Foundation of India, All India Institute of Medical Sciences, New Delhi, Madras Diabetes Research Foundation, Chennai, Aga Khan University, Karachi and Rollins School of Public Health, Emory University, USA. Study participants provided written informed consent, before participation in the study.

Analysis

We used Stata (version 14.0 SE; StataCorp, TX, USA) for data analysis. We used the 'svy' command for all analysis to account for the complex survey design (20). Before any of the survey estimation commands were used, the svyset command was used to specify the variables that described the stratification, sampling weight, and primary sampling unit variables. This analysis included data obtained from 16,284 study participants. All the responses coded as refused, unknown, or missing were treated as missing data. The frequency (percentages) and mean were reported to display the level of population health status and the sample characteristics. Percentages of those reporting any problems in EQ5D domains and mean VAS were stratified by respondent's demographic characteristics - age, gender, marital status, and SES - education, income, and employment status; and health-related indicators - presence of chronic conditions, were reported. Additionally, prevalence-ratios of moderate or severe health problems in people with and without chronic conditions were estimated using log binomial regression. Where the model did not reach convergence Poisson regression model was used. The model was adjusted for socio-demographic covariates (age, gender, marital status, education level, and household income) and city. Linear regression analysis was performed to explore the relationship between the VAS and the EQ5D measures across

For peer review only - http://bmjopen.bmj.com/site/about/guidelines.xhtml

major subgroups. In the regression model, VAS was used as a dependent variable, and EQ5D measures were treated as independent variables.

Study Results

Characteristics of the study population

A total of 17,274 individuals in 10,002 households were approached in the three study sites (7,596 participants in Chennai, 5,420 in Delhi, 4,258 in Karachi). From these, a total of 16,287 participants were recruited (the overall response rate was 94.3% at the participant level; 6,906 Chennai [90.9%], 5,364 Delhi [98.9%], and 4,017 Karachi [94.3%]). Detailed baseline characteristics of the CARRS cohort is published elsewhere (21-24). Briefly, mean age was 42.4 (+/- 13.3), 52.4% were females, 61% completed secondary schooling, and the majority of respondents (72.5%) reported household income level <INR. 10,000 (US$200). Two-third (66%) of the study population had BMI ≥25, and one-fifth (20%) of the respondents reported current tobacco use and 37.5% had self-reported chronic conditions (hypertension, diabetes, heart disease, stroke or chronic kidney disease).

Overall HRQOL by age and gender

A total of 16,284 study participants completed the EQ5D-VAS [99.9%]. Overall, the percentage of respondents reporting any problems in mobility and pain/discomfort (14% each) were higher than for other domains. Greater health problems were observed with higher age for both men and women (p-value <0.001). [Table 1]. Problems with mobility were higher with advancing age. However, problems with anxiety/depression did not show such trend. Average health status (VAS) reported by the CARRS cohort was 74.5 (95% CI 73.7 – 74.2). [Figure 1]. Women reported lower health status than men (71.6 vs.79.0; p-value<0.001).

For peer review only - http://bmjopen.bmj.com/site/about/guidelines.xhtml

74% of the respondents rated a perfect health profile with no difficulties in any EQ-5D domain, and 0.06% rated the worst health profile whereby they had difficulties with every EQ-5D domain. The distribution of the VAS scores was skewed in the direction of best-imagined health state. Only 0.5% respondents rated their health status on VAS under 10, and 10% rated it under 50 [Appendix 1].

HRQOL and socio-economic status

Table 2 and Figure 2 depicts the mean VAS, percentage, and prevalence ratios of respondents reporting moderate or severe problems in the five dimensions, across various sub-groups, respectively. Employed adults and students reported better health status than homemakers, retired, or unemployed participants. We observed almost equal health status in homemakers and retired people. Health status was also similar in the middle- and high-income groups, while it was significantly lower in the low-income group. Individuals with higher education (graduate and above) and high income had higher HRQOL than those with secondary or primary schooling and low-income class. Also, individuals with BMI ≥18-24 Kg/m^2 reported better health status, than those with BMI ≥25 Kg/m^2. Current tobacco users reported better health status than former tobacco users or non-users. However, in a stratified analysis of HRQOL in tobacco users by presence or absence of chronic conditions, tobacco users with chronic conditions reported worse health status than non-users.

HRQOL and chronic conditions

Overall, individuals with chronic conditions reported lower health status than those without chronic conditions. About half of the respondents with self-reported diabetes, hypertension, stroke, heart disease, or chronic kidney disease reported moderate or severe problems in all five domains (Table 2).

Table 3 presents the adjusted prevalence ratio of moderate or severe problems among people with versus without chronic conditions, stratified by sex and cities. Individuals

For peer review only - http://bmjopen.bmj.com/site/about/guidelines.xhtml

with chronic conditions reported two times greater problems in mobility, usual activities domains, pain/discomfort, and anxiety/depression, than in individuals without chronic conditions.

Further, a small proportion of individuals with chronic conditions, mostly those with hypertension (10.5%) or diabetes (8.3%) reported having a perfect health state.

Relationship between VAS and EQ5D measures across major sub-groups

We expected that each EQ5D dimension would have an independent relationship with VAS since each of them represents a different aspect of HRQOL. Appendix 2 provides the beta coefficients of the weighted regression models (i.e., with the application of the population sampling weights). In the overall population, having any problems in mobility, self-care, pain/discomfort, and anxiety/depression were associated with VAS scores that were 10-12 points lower. This inverse relationship of lower VAS with higher domain difficulties was larger in men, elderly (>60 years), low-income, less educated, divorced, and high BMI individuals, compared to their respective counterparts. Tobacco users who reported difficulties in all domains of EQ5D had lower VAS scores (indicating lower quality of life). Kidney disease and stroke were the most disabling conditions on all measures.

Discussion

Comparative assessments of HRQOL variations by socio-demographic factors and chronic conditions aid in prioritizing public health targets for intervention. Results from this study indicate that less than 10% of the respondents rated their health status as 100 (i.e. best imagined health state) on VAS. Mobility, pain/discomfort, and anxiety /depression were the most commonly reported problems, with the extent of these problems differing across population subgroup. Elderly (>60 years) and women

For peer review only - http://bmjopen.bmj.com/site/about/guidelines.xhtml

reported significantly greater problems in the mobility, pain/discomfort and anxiety/depression domains.

The mean VAS in our study was 74.5, which is lower than reported by most western countries (82.5), but comparable to the results from other low- and middle-income countries (LMIC) (71.1 - 77.8) [Appendix 3] (25-28). Lower health status reported by urban South Asians can be interpreted in a number of ways. The lower scores may be related to generally lower reporting of HRQOL among this group. Alternatively, these scores may reflect morbidity and sub-optimal access to healthcare facilities to address health concerns. South Asians experience chronic conditions at relatively younger ages than other race/ethnic groups and the associated reductions in productivity and income levels may be manifested in these self-reported EQ5D-VAS scores.

A higher percentage of individuals reported problems in mobility dimension (14-17%) in South Asia, which is comparable to results from other LMIC (29). However, problems in anxiety/depression are pretty low in South Asia when compared to the rest of the world; this could be due to under diagnoses of depression related problems or stigma attached to these health conditions. These patterns could also reflect differences in how adults in different parts of the world self-rate their health.

Worse health status in retired or homemakers, compared to employed persons may be related to being homebound or reflect underlying illnesses that may be the factor driving these participants to be homebound and not employed.

In terms of modifiable risk factors, maintaining a healthy BMI cut-off (18-25 Kg/m^2) is favoured because, individuals with BMI <18 Kg/m^2 and ≥25 Kg/m^2, reported greater problems in all five domains. Although, previous studies have shown that lower levels (intensity) of tobacco use are linked with higher HRQOL and regular tobacco users with

For peer review only - http://bmjopen.bmj.com/site/about/guidelines.xhtml

worse health status (30, 31), in our study, former tobacco users reported lower HRQOL than current users. This finding may indicate reverse causality, i.e. former tobacco users after experiencing an illness would have quit smoking/tobacco. Further, supported by the fact that tobacco users with chronic conditions or greater difficulties in EQ5D domains had lower VAS scores is suggestive that morbidity and not the habit of tobacco use per se are more closely related to participants' perception of health. However, a causal link between tobacco use and HRQOL cannot be confirmed in this cross-sectional study. Longitudinal analyses of the independent associations between the smoking/tobacco with HRQOL may provide a better understanding of this relationship.

Notably, one in five individuals living with known hypertension or diabetes (average disease duration four years) still reported a perfect health state, indicating that these individuals may feel asymptomatic until they experience a clinical event. Also, very small proportions of patients with heart disease and stroke (with longer duration of illness; average nine years), reported perfect health states, suggesting that these individuals may have adapted to their conditions over time and maybe benefiting from treatment and self-care that improves their self-rated quality of life. However, we did not investigate whether these other factors like adherence influence quality life in those living with chronic conditions.

Due to the differences in statistical analyses, HRQOL measures, socio-demographic characteristics of the sample, and medical conditions selected, the results of this study may not be directly comparable to reports from other countries (32). Nevertheless, a few differences and common findings are noteworthy. Individuals with stroke or chronic kidney disease rated the lowest health status, which is consistent with results reported from other studies done in China, Thailand, and Western populations (28, 33-36). Since the respondent's health status could be affected by how well the condition

For peer review only - http://bmjopen.bmj.com/site/about/guidelines.xhtml

was managed, caution is needed in interpreting study results regarding the relative effect of chronic conditions on HRQOL (37-41). A more recent Canadian study conducted by Mo *et al*. indicated a strong relationship between low health utility index (HUI) scores and certain chronic conditions (42). The authors found that arthritis/ rheumatism, heart disease, hypertension, cataracts, and diabetes had a negative impact on HRQOL. In the US, Medical Expenditure Panel Survey data based study reported that, after adjusting for socio-demographic variables, all of the selected chronic conditions were associated with lower EQ5D scores, with effects greatest for emphysema, followed by heart disease, stroke, high BP, diabetes, and asthma(43, 44).

Strengths and limitations of this study

To our knowledge, this is the first population level HRQOL data from South Asia using EQ5D-VAS including three large metropolitan cities in India and Pakistan with a large sample size that has used multistage cluster random sampling strategy and standardized protocols and measurement tools across sites. Our data provide the first baseline values to be used for monitoring population health status and analysed the relationships between selected chronic conditions and HRQOL. This information could be used to complement national targets by providing a measure of chronic disease burden based on perceived health status rather than solely on mortality and disease prevalence. In our secondary data analysis, EQ5D and VAS measures correlated well, which confirms the convergent and discriminate validity of the EQ5D instrument.

There are several limitations to this study. First, due to the cross-sectional nature of the data, the causal relationship between socio-economic parameters/chronic conditions and HRQOL cannot be determined and is not implied. Second, many chronic conditions (respiratory, locomotor, cancer, etc.) were not included in the survey. Therefore, the ranking of most severe health conditions and associated HRQOL is not complete. Third,

For peer review only - http://bmjopen.bmj.com/site/about/guidelines.xhtml

the selected chronic conditions were self-reported, and the study investigators did not examine the accuracy of information. However, this poses less of a threat to validity because self-reporting of heart diseases, stroke, and kidney diseases are pretty accurate in community surveys (45-48). Further, hypertension and diabetes were measured in this study using standardized methods. Lastly, EQ5D data were self-reported and the variation in how individuals perceive disability varies widely. However, this should be less of a problem given the large sample size in this study. Fourth, the findings of this study may not be replicable if researchers use a different HRQOL instrument (49-54), which can be tested in a future study.

Public health relevance and Policy implications

HRQOL data from this study provide baseline values for monitoring variations in health for specific population groups on the basis of gender, education, employment, income, presence of chronic conditions and place of residence. This data is also relevant to assess the overall burden of physical and mental health problems that are not disease-specific. In aggregate form, such information could be used to complement national health targets by providing a measure based on health status (quality of life) rather than mortality or disease prevalence alone. Therefore, the policy makers can use the HRQOL measures and resulting data form this study to minimise health disparities and allocate resources among competing health programs based on burden of physical or mental health problems in a specific group(55).

The lower health status reported by females, less educated, unemployed, and low-income groups may indicate higher levels of stress in these groups (17). Other potential contributing factors that are known to influence health status are living conditions, gross domestic product (GDP) per capita, inequities in income distributions,

For peer review only - http://bmjopen.bmj.com/site/about/guidelines.xhtml

For peer review only

and access to healthcare (56-61). Therefore, public health initiatives should focus on inter-sectoral approaches to address issues of education, generating more avenues for employment, and improving the quality and access of primary healthcare.

Lastly, the issue of 'clinical' or 'policy' relevance of the difference in EQ5D measures needs much discourse. For example, if the VAS in two groups of the population is 5 or 10 points different from each other, we cannot make a clinical judgment on how much these two groups would differ in their actual health status. These issues relate to determining a minimally significant difference/change in HRQOL and needs investigation in future studies. However, because of HRQOL sensitivity to time trends as shown in previous studies(62-64), these measures are also likely to be useful in determining the effect of major population-based policies or interventions.

Conclusion

HRQOL appears to be lower with higher age and among women in South Asia. Our data demonstrates significantly lower HRQOL in key demographic groups and those with chronic conditions, which are consistent with previous studies. These data provide insights on inequalities in population health status, and potentially reveal unmet needs in the community to guide health policies.

Funding: The CARRS Study was funded in whole or in part by the National Heart, Lung, and Blood Institute of the National Institutes of Health, Department of Health and Human Services (contract no. HHSN268200900026C) and the United Health Group (Minneapolis, MN, USA). Roopa Shivashankar is supported by a Wellcome Trust Capacity Strengthening Strategic Award Extension phase to the Public Health Foundation of India and a consortium of UK universities (WT084754/Z/08/A).

For peer review only - http://bmjopen.bmj.com/site/about/guidelines.xhtml

Conflict of Interest: All authors [KS, DK, RS, MKA, RP, VSA, VM, MMK, MDS, NT KMVN, and DP) declare that they have no conflict of interest to report.

Author's contribution: KS, DP, MKA, RS, NT and KMVN conceptualized and designed the study. KS wrote the first draft of the manuscript. KS and DK performed statistical analysis. RS, VSA, MKA, RP, VM, MMK, MDS, NT, KMVN, and DP contributed significantly in the revision of the manuscript. All authors have approved the submission of this version of the manuscript.

Data sharing statement: KS, DK, and DP have access to study dataset and statistical code. Any request for data sharing should be addressed to the corresponding author (DP).

For peer review only

Tables

Table 1 - Percentage of respondents reporting moderate or severe problems in EQ5D domains, stratified by age and gender

EQ5D Dimensions	20-24 years	25-34 years	35-44 years	45-54 years	55-64 years	65-74 years	≥75 years	Overall
**Overall (N)**	1179	3752	4672	3539	2005	878	262	16287
Males (N)	591	1614	2128	1723	1026	500	178	7760
Females (N)	588	2138	2544	1816	979	378	84	8527
**Mobility**								
All respondents (%)	5.3	8.3	13.6	18.1	23.5	31.2	39	14.6
95% CI	[4.0,7.0]	[6.8,10.0]	[11.7,15.9]	[16.1,20.3]	[20.8,26.4]	[27.5,35.2]	[32.7,45.6]	[13.3,15.9]
Male (%)	2.9	3.6	6	8.7	17	20.5	34.7	8.2
95% CI	[1.5,5.6]	[2.6,5.0]	[4.8,7.3]	[7.2,10.4]	[14.1,20.4]	[16.7,24.9]	[27.5,42.7]	[7.3,9.2]
Female (%)	7.8	11.8	20.1	26.9	30.6	45.6	48	20.3
95% CI	[5.9,10.2]	[9.7,14.4]	[17.1,23.4]	[24.0,29.9]	[26.5,35.1]	[40.8,50.6]	[36.2,60.1]	[18.5,22.3]
**Self-care**								
All respondents (%)	1.6	2.6	3.8	4.7	6.9	9	14.6	4.2
95% CI	[1.0,2.8]	[2.0,3.5]	[2.9,5.0]	[3.8,5.7]	[5.4,8.8]	[6.9,11.8]	[10.3,20.2]	[3.6,4.9]
Male (%)	1.2	1.5	1.7	2.7	5.1	5.2	14	2.6
95% CI	[0.4,3.6]	[0.8,2.5]	[1.0,2.9]	[1.8,3.9]	[3.1,8.2]	[3.3,8.0]	[8.9,21.5]	[2.0,3.3]
Female (%)	2.1	3.5	5.5	6.5	8.9	14.2	15.6	5.6
95% CI	[1.2,3.5]	[2.5,5.0]	[4.1,7.5]	[5.2,8.2]	[6.7,11.6]	[10.6,18.7]	[9.4,24.9]	[4.6,6.8]
**Usual activities**								
All respondents (%)	2	3.5	4.8	7.1	10.7	16.6	23.1	6.0
95% CI	[1.2,3.2]	[2.8,4.4]	[3.8,5.9]	[6.0,8.4]	[8.9,12.8]	[13.9,19.7]	[17.8,29.4]	[5.4,6.8]
Male (%)	1.3	1.4	1.8	3.2	6.4	11	19.7	3.2
95% CI	[0.5,3.7]	[0.9,2.1]	[1.2,2.7]	[2.3,4.3]	[4.8,8.6]	[7.9,15.2]	[13.8,27.2]	[2.7,3.9]
Female (%)	2.6	5.1	7.2	10.7	15.4	24.1	30.3	8.5
95% CI	[1.6,4.3]	[4.0,6.6]	[5.6,9.2]	[8.8,12.9]	[12.3,19.1]	[20.4,28.1]	[20.7,42.0]	[7.4,9.7]
**Pain/Discomfort**								
All respondents (%)	6	9.2	13.4	18.6	20.4	27.1	30.3	14.3
95% CI	[4.4,8.1]	[7.7,10.9]	[11.5,15.5]	[16.6,20.7]	[17.9,23.1]	[23.3,31.2]	[24.1,37.2]	[13.0,15.6]
Male (%)	3.6	5.5	7.6	10.6	14.1	16.5	28	8.9
95% CI	[2.3,5.7]	[4.0,7.3]	[6.0,9.6]	[9.1,12.4]	[11.4,17.4]	[13.1,20.7]	[20.7,36.7]	[8.0,9.9]
Female (%)	8.5	11.9	18.2	25.9	27.2	41.2	35.1	19.1
95% CI	[5.7,12.3]	[9.8,14.5]	[15.5,21.3]	[23.0,29.1]	[23.5,31.1]	[35.6,47.0]	[25.0,46.7]	[17.1,21.2]
**Anxiety/Depression**								
All respondents (%)	4.9	5.8	7.6	9.9	10.9	13.7	18	8.1
95% CI	[3.7,6.6]	[4.8,7.0]	[6.5,8.9]	[8.7,11.4]	[9.4,12.7]	[11.2,16.7]	[13.3,23.7]	[7.4,8.9]
Male (%)	3.7	4.1	5.4	6.7	8.7	7.6	15.7	5.9
95% CI	[2.2,6.0]	[3.1,5.5]	[4.3,6.8]	[5.2,8.5]	[6.8,11.0]	[5.4,10.7]	[10.7,22.6]	[5.2,6.7]
Female (%)	6.2	7.0	9.5	13	13.4	21.9	22.7	10.1
95% CI	[4.4,8.6]	[5.6,8.8]	[7.9,11.4]	[11.0,15.2]	[11.1,16.1]	[18.0,26.5]	[14.0,34.6]	[9.1,11.3]

Abbreviations: CI – confidence interval, EQ5D – European Quality of Life 5 dimension, N – number of participants

For peer review only - http://bmjopen.bmj.com/site/about/guidelines.xhtml

Figure 1 - Mean self-rated health status using EQ5D-VAS of respondents by age groups and gender

Figure Legend: This figure presents the mean self-rated health status for overall study population by age-groups and gender. European Quality of Life 5 Dimension - Visual Analogue Scale (EQ5D-VAS) measures health status on a scale of 0 (worst health status) – 100 (best imaginable health status)

*** P-value for difference between mean EQ5D-VAS between males and females at each age-group is statistically significant; p<0.01.**

For peer review only

For peer review only - http://bmjopen.bmj.com/site/about/guidelines.xhtml

Figure 2 – Prevalence ratio of moderate or severe health problems by socio-demographic factors and chronic conditions

Figure 2.a. shows the prevalence ratio of moderate or severe difficulties in EQ5D domains (mobility, self-care, usual activities, pain/discomfort, anxiety/depression and any of the five dimensions) by employment status. With reference to those who were employed (PR=1), housewife, retired, and unemployed reported greater problems in all five domains. Whereas, students only reported higher anxiety problems compared to employed.

Figure 2.b. shows the prevalence ratio of moderate or severe difficulties in EQ5D domains by income-group. With reference to low-income group (PR=1), those in middle- or high-income groups had less problems in all five domains.

Figure 2.c. shows the prevalence ratio of moderate or severe difficulties in EQ5D domains (mobility, self-care, usual activities, pain/discomfort, anxiety/depression and any of the five dimensions) by education level. With reference to those primary school education (PR=1), individuals with secondary school or graduates reported significantly lower problems in all five domains.

Figure 2.d. shows the prevalence ratio of moderate or severe difficulties in EQ5D domains by marital status. With reference to single (PR=1), those who were married, widower, or divorcee had greater problems in all five domains.

Figure 2.e. shows the prevalence ratio of moderate or severe difficulties in EQ5D domains (mobility, self-care, usual activities, pain/discomfort, anxiety/depression and any of the five dimensions) by body mass index (BMI). With reference to underweight i.e. BMI $<18 Kg/m^2$ (PR=1), individuals with overweight (BMI 25-29.9 Kg/m^2) or obesity (BMI$\geq$30Kg/m^2) reported significantly greater problems in all five domains.

Figure 2.f. shows the prevalence ratio of moderate or severe difficulties in EQ5D domains by chronic conditions. Compared to those without chronic conditions, individuals with self-reported diabetes, hypertension, heart disease, stroke and kidney disease had twice greater problems in all five domains.

Abbreviations: *AD – Any Dimension, HRQOL – Health-related quality of life, SC – Self-Care, UA – Usual Activities; PR – Prevalence Ratio, EQ5D – European Quality of Life 5 dimension*

For peer review only - http://bmjopen.bmj.com/site/about/guidelines.xhtml

Table 2 - Mean EQ-VAS and percentages of respondents reporting moderate or severe problems by various subgroups

	No. of respondents	EQ-VAS Mean	95%CI	Mobility (%)	95%CI	Self-care (%)	95%CI	Usual activities (%)	95%CI	Pain/discomfort (%)	95%CI	Anxiety/ depression (%)	95%CI
**Cities**													
Chennai	6906	70.7	[70.1,71.4]	17.3	[15.4,19.4]	7.8	[6.5,9.2]	7.7	[6.6,9.0]	10.3	[9.1,11.7]	11.4	[9.8,13.1]
Delhi	5364	78.8	[77.8,79.8]	14.1	[11.9,16.7]	1.6	[1.2,2.1]	4.4	[3.4,5.7]	19.4	[16.9,22.2]	5.2	[4.0,6.6]
Karachi	4017	73.2	[72.6,73.8]	10.4	[9.4,11.5]	1.9	[1.5,2.4]	5.6	[4.9,6.4]	12.9	[11.8,14.2]	7.2	[6.3,8.1]
**Employment status**													
Employed	7635	77.2	[76.4,78.1]	8.7	[7.7,9.9]	2.4	[1.8,3.1]	2.8	[2.3,3.4]	9.3	[8.3,10.4]	6.2	[5.4,7.1]
Student	361	77.5	[75.2,79.7]	6.5	[4.1,10.0]	1.5	[0.7,3.4]	2.6	[1.3,4.9]	8.3	[5.4,12.7]	7.0	[4.4,10.8]
Homemakers	6781	71.6	[70.7,72.4]	20.9	[18.9,22.9]	5.7	[4.8,6.8]	9.0	[7.8,10.4]	19.7	[17.6,22.0]	9.9	[8.9,11.0]
Retired	765	71.6	[69.9,73.3]	21.0	[17.7,24.6]	7.7	[5.7,10.3]	11.0	[8.5,14.0]	18.1	[15.0,21.8]	9.0	[7.0,11.5]
Unemployed	743	68.3	[66.8,69.9]	17.1	[13.6,21.2]	7.7	[5.7,10.5]	9.5	[7.3,12.4]	16.1	[13.2,19.6]	12.3	[9.8,15.2]
**Income class**													
Low income group (INR <10000 or US$ 155)	11537	73.4	[72.7,74.1]	15.2	[13.9,16.7]	4.8	[4.1,5.7]	6.8	[6.0,7.6]	14	[12.7,15.4]	9.2	[8.3,10.1]
Middle income group (INR 10000-20000 or US$ 155-310)	2667	75.8	[74.8,76.9]	14.6	[12.6,16.8]	3.5	[2.6,4.7]	4.9	[3.9,6.1]	14.5	[12.4,17.0]	6.4	[5.3,7.6]
High income group (INR >20000 or US$>310)	1975	77.1	[75.9,78.3]	11.0	[8.8,13.8]	1.5	[1.0,2.2]	3.4	[2.3,4.9]	15.5	[12.8,18.6]	5.0	[3.9,6.4]
**Education status**													
Up to primary school	3604	71.1	[70.1,72.2]	21.6	[19.5,24.0]	5.6	[4.7,6.7]	9.8	[8.5,11.2]	20.8	[18.5,23.3]	10.9	[9.6,12.4]
Secondary school	9924	74.3	[73.5,75.1]	14.0	[12.6,15.5]	4.5	[3.7,5.3]	5.8	[5.1,6.7]	13.0	[11.8,14.4]	8.1	[7.3,9.0]
Graduation and above	2759	77.9	[76.9,78.8]	8.3	[6.7,10.2]	1.6	[1.1,2.2]	2.3	[1.7,3.1]	10.8	[9.0,12.8]	5.0	[4.1,6.0]
**Marital Status**													
Single	1177	78.2	[76.9,79.6]	6.7	[5.2,8.7]	1.6	[0.9,2.9]	2.0	[1.2,3.2]	7.1	[5.5,9.2]	5.4	[3.9,7.5]
Married	14217	74.3	[73.6,75.0]	14.2	[13.0,15.6]	4.1	[3.5,4.8]	5.7	[5.0,6.5]	14	[12.6,15.4]	7.8	[7.1,8.6]

For peer review only - http://bmjopen.bmj.com/site/about/guidelines.xhtml

Widowed	838	67.4	[66.1,68.7]	34.5	[30.5,38.8]	10.2	[8.0,12.9]	18.7	[15.8,22.1]	32.7	[29.3,36.2]	18.2	[15.4,21.3]
Separated/Divorced	55	65.3	[57.9,72.7]	24.6	[11.8,44.3]	10.2	[4.6,21.3]	20.6	[8.8,41.0]	22.3	[12.2,37.2]	25.1	[14.9,39.2]
**BMI (Kg/m^2)**													
underweight (<18)	756	74.0	[72.3,75.7]	14.9	[11.7,18.8]	3.0	[1.7,5.3]	5.4	[3.7,7.9]	12.9	[10.1,16.5]	9.0	[6.3,12.7]
Normal (18-24.9)	5278	75.1	[74.3,75.9]	11.8	[10.4,13.3]	3.5	[2.8,4.2]	4.8	[4.0,5.7]	11.5	[10.2,13.0]	7.5	[6.6,8.7]
Overweight (25.0-29.9)	4190	73.6	[72.7,74.5]	15.2	[13.4,17.2]	4.8	[3.8,6.0]	6.2	[5.2,7.3]	14.4	[12.6,16.4]	8.2	[7.2,9.4]
Obesity (≥30)	2249	70.4	[69.5,71.2]	22.3	[19.9,24.9]	6.4	[5.1,8.0]	9.1	[7.6,10.9]	20.7	[18.2,23.6]	10	[8.5,11.8]
**Tobacco use (Smoke/Chew/other forms)**													
Never user	12215	74.1	[73.4,74.8]	15.4	[14.0,16.8]	4.5	[3.8,5.3]	6.4	[5.6,7.2]	14.6	[13.1,16.2]	8.1	[7.3,9.0]
Current user	3758	75.3	[74.3,76.3]	11.8	[10.4,13.4]	3.1	[2.4,4.1]	4.6	[3.8,5.6]	12.9	[11.6,14.5]	7.9	[6.7,9.2]
Former user	314	70.1	[67.0,73.2]	17.6	[13.4,22.8]	4.7	[2.7,8.1]	8.3	[5.6,12.3]	18.1	[13.6,23.7]	12.4	[8.8,17.2]
**Chronic conditions (self-reported)**													
No	12498	76.2	[75.4,76.9]	11.9	[10.7,13.1]	3.5	[2.8,4.2]	4.5	[3.9,5.2]	11.2	[10.1,12.5]	6.8	[6.0,7.6]
Yes	4699	67.3	[66.6,68.1]	24.6	[22.4,27.0]	6.9	[5.9,7.9]	11.8	[10.5,13.2]	25.5	[23.3,27.9]	13.2	[12.0,14.5]
**Diabetes**													
No diabetes	4610	75.1	[74.4,75.9]	10.1	[8.9,11.4]	3.3	[2.6,4.1]	4.2	[3.5,5.0]	10.3	[8.8,12.0]	7.5	[6.5,8.6]
Pre-diabetes	5449	74.4	[73.5,75.3]	15.5	[13.6,17.5]	4.2	[3.3,5.2]	6.0	[5.0,7.2]	14.6	[12.9,16.4]	8.0	[7.0,9.1]
Newly diagnosed	2015	74.3	[73.3,75.3]	17.2	[14.7,20.1]	5.0	[3.8,6.5]	8.1	[6.5,10.0]	17.2	[15.0,19.6]	8.5	[7.1,10.1]
Self-reported Diabetes	1661	65.9	[64.8,67.1]	20.9	[18.9,23.1]	6.3	[5.2,7.5]	9.5	[8.3,11.0]	19.8	[18.1,21.7]	10.5	[9.3,11.8]
**Hypertension**													
Normotension	5695	74.8	[74.0,75.5]	12.8	[11.4,14.3]	3.8	[3.0,4.9]	4.7	[3.9,5.6]	11.6	[9.9,13.6]	6.9	[6.0,8.0]
Prehypertension	4717	76.0	[75.0,76.9]	12.7	[11.2,14.4]	3.7	[2.9,4.6]	5.1	[4.3,6.0]	12.3	[11.0,13.8]	6.5	[5.6,7.5]
Newly diagnosed	2780	75.8	[74.8,76.8]	12.9	[11.0,15.1]	3.0	[2.4,3.8]	5.1	[4.2,6.3]	12.6	[11.0,14.3]	8.4	[7.2,9.9]
Self-reported	2397	66.7	[65.8,67.7]	18.8	[16.8,21.0]	4.9	[4.2,5.7]	8.7	[7.6,9.9]	19.6	[17.8,21.4]	10.8	[9.7,12.0]
**Heart Disease**													
No	15842	74.6	[73.9,75.2]	14.2	[12.9,15.5]	4.0	[3.4,4.7]	5.6	[5.0,6.4]	13.8	[12.6,15.2]	7.9	[7.2,8.6]
Yes	445	63.3	[61.3,65.2]	31.2	[25.7,37.3]	11.7	[8.3,16.2]	20.8	[16.7,25.7]	31.4	[26.5,36.7]	19.0	[15.0,23.8]
**Stroke**													
No	16203	74.3	[73.6,75.1]	14.5	[13.3,15.8]	4.1	[3.5,4.8]	6.0	[5.3,6.7]	14.1	[12.9,15.5]	8.1	[7.4,8.8]
Yes	84	62.4	[58.5,66.2]	31.9	[22.0,43.7]	16.8	[10.0,26.7]	18.1	[11.1,28.1]	43.3	[32.3,55.1]	21.2	[13.2,32.2]

For peer review only

For peer review only - http://bmjopen.bmj.com/site/about/guidelines.xhtml

**Kidney disease**														
	No	16175	74.4	[73.7,75.1]	14.6	[13.3,15.9]	4.1	[3.6,4.8]	6.0	[5.3,6.7]	14.1	[12.9,15.5]	8.1	[7.3,8.8]
	Yes	112	62.5	[59.4,65.7]	19.2	[13.1,27.2]	10	[5.7,17.0]	15.1	[9.9,22.3]	31.4	[23.5,40.6]	20.7	[14.1,29.3]

NOTES: Tobacco use, Heart disease, Kidney disease were based on self-reports, newly diagnosed diabetes - defined as no self-reported diabetes and fasting blood glucose (FBG) of ≥126 mg/dl, or HbA1c ≥6.5%), pre-diabetes – no self-reported diabetes and FBG ≥100-125 mg/dl or HbA1c ≥5.7-6.4%), normoglycemia –no self-reported diabetes and FBG<100 mg/dl and HbA1c<5.7%, Newly diagnosed hypertension – defined as no self-reported hypertension and BP ≥140/90mmHg, prehypertension - no self-reported hypertension and BP: 120-139 / 80-89 mmHg and normotensive - no history of hypertension and BP <120/80 mmHg.

Abbreviations: *INR: Indian rupees, mmHg – millimetre of mercury, mg/dl - Milligram/decilitre; US$ - United States Dollar, CI – Confidence Interval*

For peer review only

Table 3: Adjusted prevalence ratio of reporting any problems in individuals with chronic conditions versus those without chronic conditions, by cities and gender

	Mobility		Self-care		Usual activities		Pain/discomfort		Anxiety/depression		Any Dimension	
	PR	95%CI	PR	95%CI	PR	95%CI	PR	95%CI	PR	95%CI	PR	95%CI
**Overall**$	1.41	[1.27,1.57]	1.35	[1.09,1.67]	1.70	[1.45,2.01]	1.74	[1.58,1.91]	1.60	[1.40,1.84]	1.45	[1.35,1.56]
Males^	1.78	[1.46,2.18]	1.60	[1.10,2.34]	2.01	[1.41,2.87]	1.89	[1.62,2.23]	2.01	[1.55,2.62]	1.63	[1.41,1.88]
Females^	1.32	[1.16,1.50]	1.28	[0.99,1.66]	1.67	[1.40,1.99]	1.72	[1.53,1.93]	1.44	[1.23,1.69]	1.39	[1.29,1.52]
**Chennai***	1.24	[1.05,1.46]	1.08	[0.84,1.40]	1.31	[1.07,1.60]	1.71	[1.45,2.04]	1.56	[1.31,1.86]	1.30	[1.17,1.44]
Males^	1.46	[1.07,1.99]	1.12	[0.71,1.76]	1.24	[0.80,1.90]	1.73	[1.34,2.24]	1.75	[1.34,2.30]	1.38	[1.16,1.66]
Females^	1.18	[0.97,1.44]	1.08	[0.79,1.47]	1.38	[1.11,1.73]	1.73	[1.38,2.17]	1.46	[1.16,1.83]	1.27	[1.12,1.45]
**Delhi***	1.66	[1.41,1.95]	3.05	[1.78, 5.25]	2.40	[1.66,3.47]	1.79	[1.54,2.08]	1.84	[1.29,2.63]	1.65	[1.47,1.86]
Males^	2.13	[1.52,2.97]	5.14	[1.92,13.78]	3.63	[1.61,8.17]	1.93	[1.50,2.49]	3.45	[1.36,8.80]	1.89	[1.45,2.48]
Females^	1.58	[1.31,1.90]	2.23	[1.27,3.94]	2.15	[2.15,2.15]	1.82	[1.51,2.18]	1.56	[1.12,2.18]	1.62	[1.43,1.83]
**Karachi***	1.41	[1.15,1.72]	1.77	[1.05,3.00]	2.09	[1.58,2.77]	1.64	[1.37,1.97]	1.60	[1.21,2.11]	1.43	[1.25,1.65]
Males^	2.51	[1.52,4.13]	1.76	[0.77,4.02]	2.91	[1.62,5.22]	2.28	[1.52,3.42]	2.04	[1.27,3.29]	2.01	[1.44,2.80]
Females^	1.24	[1.01,1.53]	2.11	[1.10,4.04]	1.95	[1.44,2.65]	1.50	[1.24,1.81]	1.32	[0.94,1.87]	1.27	[1.10,1.47]

NOTES: Prevalence ratio (PR) were estimated using Poisson regression model as the log binomial regression model did not reach convergence.
$adjusted for age, sex, income, education, marital status and city
*adjusted for age, sex, income, education, and marital status
^adjusted for age, income, education, marital status and city

Abbreviations: CI – Confidence interval, PR – Prevalence ratio,

For peer review only - http://bmjopen.bmj.com/site/about/guidelines.xhtml

References

1. Rabin R, de Charro F. EQ-5D: a measure of health status from the EuroQol Group. Ann Med. 2001;33(5):337-43.
2. Barker AE. Clinical Lecture on Permanent Subcutaneous Suture of the Patella for Recent Fracture: Delivered at University College Hospital. Br Med J. 1892;1(1626):425-8.
3. Kaplan RM, Anderson JP. A general health policy model: update and applications. Health Serv Res. 1988;23(2):203-35.
4. Coons SJ, Rao S, Keininger DL, Hays RD. A comparative review of generic quality-of-life instruments. Pharmacoeconomics. 2000;17(1):13-35.
5. Prabhakaran D, Jeemon P, Roy A. Cardiovascular Diseases in India: Current Epidemiology and Future Directions. Circulation. 2016;133(16):1605-20.
6. Fortin M, Lapointe L, Hudon C, Vanasse A, Ntetu AL, Maltais D. Multimorbidity and quality of life in primary care: a systematic review. Health Qual Life Outcomes. 2004;2:51.
7. Alonso J, Ferrer M, Gandek B, Ware JE, Jr., Aaronson NK, Mosconi P, et al. Health-related quality of life associated with chronic conditions in eight countries: results from the International Quality of Life Assessment (IQOLA) Project. Qual Life Res. 2004;13(2):283-98.
8. Schultz SE, Kopec JA. Impact of chronic conditions. Health Rep. 2003;14(4):41-53.
9. Patrick DL, Deyo RA. Generic and disease-specific measures in assessing health status and quality of life. Med Care. 1989;27(3 Suppl):S217-32.
10. Horsman J, Furlong W, Feeny D, Torrance G. The Health Utilities Index (HUI): concepts, measurement properties and applications. Health Qual Life Outcomes. 2003;1:54.
11. Furlong WJ, Feeny DH, Torrance GW, Barr RD. The Health Utilities Index (HUI) system for assessing health-related quality of life in clinical studies. Ann Med. 2001;33(5):375-84.
12. Brazier J, Roberts J, Deverill M. The estimation of a preference-based measure of health from the SF-36. J Health Econ. 2002;21(2):271-92.
13. McHorney CA. Health status assessment methods for adults: past accomplishments and future challenges. Annu Rev Public Health. 1999;20:309-35.
14. The World Health Organization Quality of Life Assessment (WHOQOL): development and general psychometric properties. Soc Sci Med. 1998;46(12):1569-85.
15. The World Health Organization Quality of Life assessment (WHOQOL): position paper from the World Health Organization. Soc Sci Med. 1995;41(10):1403-9.
16. Study protocol for the World Health Organization project to develop a Quality of Life assessment instrument (WHOQOL). Qual Life Res. 1993;2(2):153-9.
17. Centers for Disease Control and Prevention. Measuring healthy days: Population assessment of health-related quality of life. Centers for Disease Control and Prevention, Atlanta, Georgia 2000.
18. Gandek B, Sinclair SJ, Kosinski M, Ware JE, Jr. Psychometric evaluation of the SF-36 health survey in Medicare managed care. Health Care Financ Rev. 2004;25(4):5-25.
19. Nair M, Ali MK, Ajay VS, Shivashankar R, Mohan V, Pradeepa R, et al. CARRS Surveillance study: design and methods to assess burdens from multiple perspectives. BMC Public Health. 2012;12:701.
20. ISPOR 2nd Latin America Conference. Abstracts. Value Health. 2009;12(7):A483-534.
21. Patel SA, Shivashankar R, Ali MK, Anjana RM, Deepa M, Kapoor D, et al. Is the "South Asian Phenotype" Unique to South Asians?: Comparing Cardiometabolic Risk Factors in the CARRS and NHANES Studies. Glob Heart. 2016;11(1):89-96 e3.
22. Deepa M, Grace M, Binukumar B, Pradeepa R, Roopa S, Khan HM, et al. High burden of prediabetes and diabetes in three large cities in South Asia: The Center for cArdio-metabolic Risk Reduction in South Asia (CARRS) Study. Diabetes Res Clin Pract. 2015;110(2):172-82.
23. Berg CJ, Ajay VS, Ali MK, Kondal D, Khan HM, Shivashankar R, et al. A cross-sectional study of the prevalence and correlates of tobacco use in Chennai, Delhi, and Karachi: data from the CARRS study. BMC Public Health. 2015;15:483.
24. Ali MK, Bhaskarapillai B, Shivashankar R, Mohan D, Fatmi ZA, Pradeepa R, et al. Socioeconomic status and cardiovascular risk in urban South Asia: The CARRS Study. Eur J Prev Cardiol. 2016;23(4):408-19.

For peer review only - http://bmjopen.bmj.com/site/about/guidelines.xhtml

25. Kind P, Dolan P, Gudex C, Williams A. Variations in population health status: results from a United Kingdom national questionnaire survey. BMJ. 1998;316(7133):736-41.
26. Sun H, Zhang Q, Luo X, Quan H, Zhang F, Liu C, et al. Changes of adult population health status in China from 2003 to 2008. PLoS One. 2011;6(12):e28411.
27. Sun S, Chen J, Johannesson M, Kind P, Xu L, Zhang Y, et al. Regional differences in health status in China: population health-related quality of life results from the National Health Services Survey 2008. Health Place. 2011;17(2):671-80.
28. Sun S, Chen J, Johannesson M, Kind P, Xu L, Zhang Y, et al. Population health status in China: EQ-5D results, by age, sex and socio-economic status, from the National Health Services Survey 2008. Qual Life Res. 2011;20(3):309-20.
29. Measuring Self-Reported Population Health: An International Perspective based on EQ-5D. In: Szende A, Williams, A., ed. On behalf of: The EuroQol Group's International Task Forceon Self-Reported Health; 2004.
30. Sayed BA, French MT. To your health!: Re-examining the health benefits of moderate alcohol use. Soc Sci Med. 2016;167:20-8.
31. Mauro PM, Canham SL, Martins SS, Spira AP. Substance-use coping and self-rated health among US middle-aged and older adults. Addict Behav. 2015;42:96-100.
32. Badia X, Herdman M, Kind P. The influence of ill-health experience on the valuation of health. Pharmacoeconomics. 1998;13(6):687-96.
33. Sullivan PW, Ghushchyan V. Preference-Based EQ-5D index scores for chronic conditions in the United States. Med Decis Making. 2006;26(4):410-20.
34. Ko Y, Coons SJ. Self-reported chronic conditions and EQ-5D index scores in the US adult population. Curr Med Res Opin. 2006;22(10):2065-71.
35. Sakthong P, Sonsa-Ardjit N, Sukarnjanaset P, Munpan W. Psychometric properties of the EQ-5D-5L in Thai patients with chronic diseases. Qual Life Res. 2015;24(12):3015-22.
36. Tan Z, Liang Y, Liu S, Cao W, Tu H, Guo L, et al. Health-related quality of life as measured with EQ-5D among populations with and without specific chronic conditions: a population-based survey in Shaanxi Province, China. PLoS One. 2013;8(7):e65958.
37. McPherson K, Myers J, Taylor WJ, McNaughton HK, Weatherall M. Self-valuation and societal valuations of health state differ with disease severity in chronic and disabling conditions. Med Care. 2004;42(11):1143-51.
38. Lam JM, Wodchis WP. The relationship of 60 disease diagnoses and 15 conditions to preference-based health-related quality of life in Ontario hospital-based long-term care residents. Med Care. 2010;48(4):380-7.
39. Hutchinson AF, Graco M, Rasekaba TM, Parikh S, Berlowitz DJ, Lim WK. Relationship between health-related quality of life, comorbidities and acute health care utilisation, in adults with chronic conditions. Health Qual Life Outcomes. 2015;13:69.
40. Hevey D, McGee HM, Horgan J. Relationship of initial level of distress to changes in health-related quality of life during cardiac rehabilitation or usual care. Psychosom Med. 2007;69(8):793-7.
41. Arnlind MH, Nokela M, Rehnberg C, Jonsson EW. The relationship between pharmaceutical costs, disease severity, and health-related quality of life in asthmatics in Swedish primary care. J Asthma. 2006;43(8):585-91.
42. Mo F, Choi BC, Li FC, Merrick J. Using Health Utility Index (HUI) for measuring the impact on health-related quality of Life (HRQL) among individuals with chronic diseases. ScientificWorldJournal. 2004;4:746-57.
43. Lubetkin EI, Jia H, Franks P, Gold MR. Relationship among sociodemographic factors, clinical conditions, and health-related quality of life: examining the EQ-5D in the U.S. general population. Qual Life Res. 2005;14(10):2187-96.
44. Jia H, Lubetkin EI. The impact of obesity on health-related quality-of-life in the general adult US population. J Public Health (Oxf). 2005;27(2):156-64.
45. Wu SC, Li CY, Ke DS. The agreement between self-reporting and clinical diagnosis for selected medical conditions among the elderly in Taiwan. Public Health. 2000;114(2):137-42.

For peer review only - http://bmjopen.bmj.com/site/about/guidelines.xhtml

46. Molenaar EA, Van Ameijden EJ, Grobbee DE, Numans ME. Comparison of routine care self-reported and biometrical data on hypertension and diabetes: results of the Utrecht Health Project. Eur J Public Health. 2007;17(2):199-205.
47. Robinson JR, Young TK, Roos LL, Gelskey DE. Estimating the burden of disease. Comparing administrative data and self-reports. Med Care. 1997;35(9):932-47.
48. Okura Y, Urban LH, Mahoney DW, Jacobsen SJ, Rodeheffer RJ. Agreement between self-report questionnaires and medical record data was substantial for diabetes, hypertension, myocardial infarction and stroke but not for heart failure. J Clin Epidemiol. 2004;57(10):1096-103.
49. Chen J, Wong, C.K.H., McGhee,S.M., Pang,P.K.P., Yu, W.C. A Comparison between the EQ-5D and the SF-6D in Patients with Chronic Obstructive Pulmonary Disease (COPD). PLoS One. 2014;9(11).
50. Salaffi F, Carotti M, Ciapetti A, Gasparini S, Grassi W. A comparison of utility measurement using EQ-5D and SF-6D preference-based generic instruments in patients with rheumatoid arthritis. Clin Exp Rheumatol. 2011;29(4):661-71.
51. Wu J, Han Y, Zhao FL, Zhou J, Chen Z, Sun H. Validation and comparison of EuroQoL-5 dimension (EQ-5D) and Short Form-6 dimension (SF-6D) among stable angina patients. Health Qual Life Outcomes. 2014;12:156.
52. Petrou S, Hockley C. An investigation into the empirical validity of the EQ-5D and SF-6D based on hypothetical preferences in a general population. Health Econ. 2005;14(11):1169-89.
53. Obradovic M, Lal A, Liedgens H. Validity and responsiveness of EuroQol-5 dimension (EQ-5D) versus Short Form-6 dimension (SF-6D) questionnaire in chronic pain. Health Qual Life Outcomes. 2013;11:110.
54. Kuspinar A, Mayo NE. A review of the psychometric properties of generic utility measures in multiple sclerosis. Pharmacoeconomics. 2014;32(8):759-73.
55. Rothberg AE, McEwen LN, Kraftson AT, Neshewat GM, Fowler CE, Burant CF, et al. The impact of weight loss on health-related quality-of-life: implications for cost-effectiveness analyses. Qual Life Res. 2014;23(4):1371-6.
56. Kindig DA BB, Remington PL. Mobilizing Action Toward Community Health (MATCH): metrics, incentives, and partnerships for population health. . Prev Chronic Dis. 2010;7(5).
57. Hennessy CH, Moriarty DG, Zack MM, Scherr PA, Brackbill R. Measuring health-related quality of life for public health surveillance. Public Health Rep. 1994;109(5):665-72.
58. Dominick KL, Ahern FM, Gold CH, Heller DA. Relationship of health-related quality of life to health care utilization and mortality among older adults. Aging Clin Exp Res. 2002;14(6):499-508.
59. Prazeres F, Santiago L. Relationship between health-related quality of life, perceived family support and unmet health needs in adult patients with multimorbidity attending primary care in Portugal: a multicentre cross-sectional study. Health Qual Life Outcomes. 2016;14(1):156.
60. Polat U, Bayrak Kahraman B, Kaynak I, Gorgulu U. Relationship among health-related quality of life, depression and awareness of home care services in elderly patients. Geriatr Gerontol Int. 2016;16(11):1211-9.
61. Lim JW, Paek MS. The relationship between communication and health-related quality of life in survivorship care for Chinese-American and Korean-American breast cancer survivors. Support Care Cancer. 2013;21(4):1157-66.
62. Jia H, Lubetkin EI. Time trends and seasonal patterns of health-related quality of life among U.S. adults. Public Health Rep. 2009;124(5):692-701.
63. Ford ES, Mokdad AH, Li C, McGuire LC, Strine TW, Okoro CA, et al. Gender differences in coronary heart disease and health-related quality of life: findings from 10 states from the 2004 behavioral risk factor surveillance system. J Womens Health (Larchmt). 2008;17(5):757-68.
64. Zahran HS, Kobau R, Moriarty DG, Zack MM, Holt J, Donehoo R. Health-related quality of life surveillance--United States, 1993-2002. MMWR Surveill Summ. 2005;54(4):1-35.

For peer review only - http://bmjopen.bmj.com/site/about/guidelines.xhtml

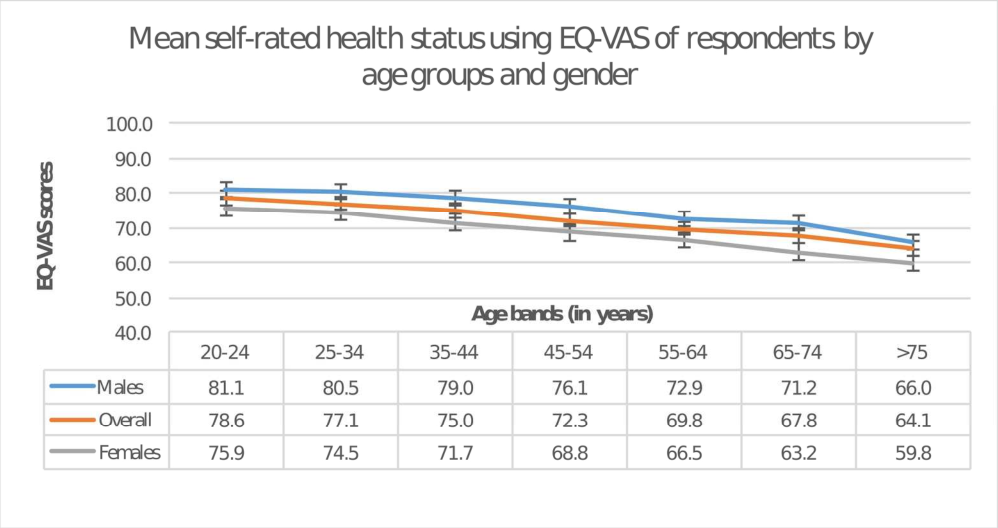

Figure 1 - Mean self-rated health status using EQ5D-VAS of respondents by age groups and gender

Figure Legend: This figure presents the mean self-rated health status for overall study population by age-groups and gender. European Quality of Life 5 Dimension - Visual Analogue Scale (EQ5D-VAS) measures health status on a scale of 0 (worst health status) – 100 (best imaginable health status)
* P-value for difference between mean EQ5D-VAS between males and females at each age-group is statistically significant; p<0.01.

88x47mm (600 x 600 DPI)

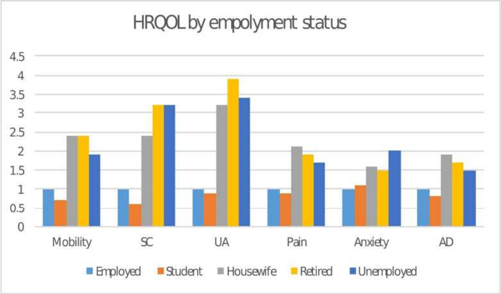

Figure 2.a

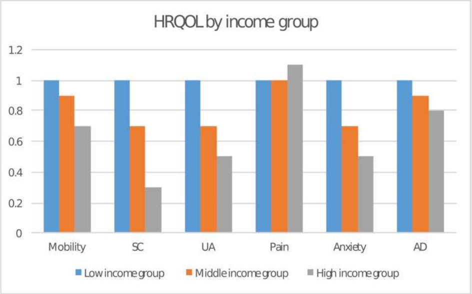

Figure 2.b

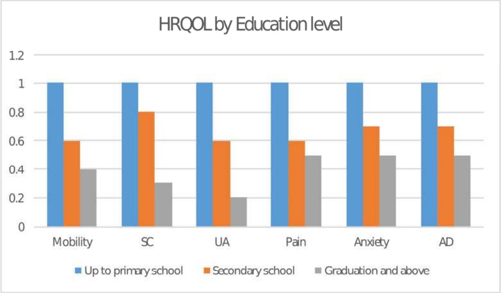

Figure 2.c

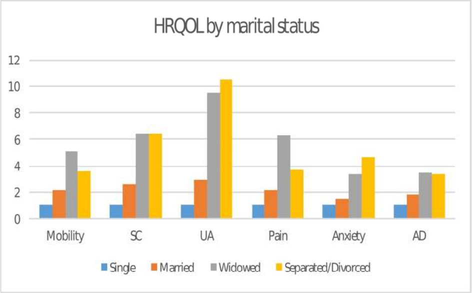

Figure 2.d

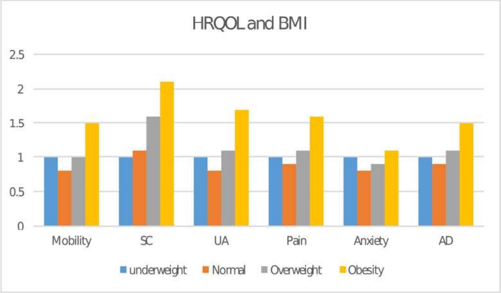

Figure 2.e

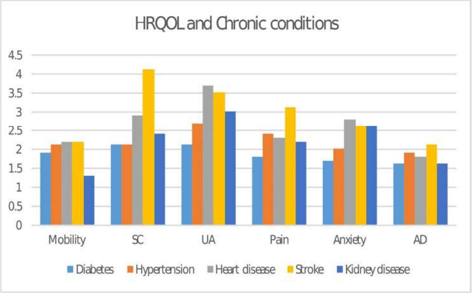

Figure 2.f

Figure 2 – Prevalence ratio of moderate or severe health problems by socio-demographic factors and chronic conditions

Figure 2.a. shows the prevalence ratio of moderate or severe difficulties in EQ5D domains (mobility, self-care, usual activities, pain/discomfort, anxiety/depression and any of the five dimensions) by employment status. With reference to those who were employed (PR=1), housewife, retired, and unemployed reported greater problems in all five domains. Whereas, students only reported higher anxiety problems compared to employed.

Figure 2.b. shows the prevalence ratio of moderate or severe difficulties in EQ5D domains by income-group. With reference to low-income group (PR=1), those in middle- or high- income groups had less problems in all five domains.

Figure 2.c. shows the prevalence ratio of moderate or severe difficulties in EQ5D domains by education level. With reference to those primary school education (PR=1), individuals with secondary school or graduates reported significantly lower problems in all five domains.

Figure 2.d. shows the prevalence ratio of moderate or severe difficulties in EQ5D domains by marital status. With reference to single (PR=1), those who were married, widower, or divorcee had greater problems in all five domains.

Figure 2.e. shows the prevalence ratio of moderate or severe difficulties in EQ5D domains by body mass index (BMI). With reference to underweight i.e. BMI <18Kg/m2 (PR=1), individuals with overweight (BMI 25-29.9 Kg/m2) or obesity (BMI≥30Kg/m2) reported significantly greater problems in all five domains.

Figure 2.f. shows the prevalence ratio of moderate or severe difficulties in EQ5D domains by chronic conditions. Compared to those without chronic conditions, individuals with self-reported diabetes, hypertension, heart disease, stroke and kidney disease had twice greater problems in all five domains. Abbreviations: AD – Any Dimension, HRQOL – Health-related quality of life, SC – Self-Care, UA – Usual Activities; PR – Prevalence Ratio, EQ5D – European Quality of Life 5 dimension

181x189mm (300 x 300 DPI)

For peer review only

Web-appendix (supplementary file)

Appendix 1: The distribution of the respondents EQ-VAS values

EQ-VAS score	Number	Percentage (%)	Cum. Percentage (%)
10-	29	0.18	0.18
20-	72	0.44	0.62
30-	88	0.54	1.16
40-	365	2.24	3.4
50-	2,056	12.62	16.03
60-	1,979	12.15	28.18
70-	3,728	22.89	51.07
80-	4,026	24.72	75.78
90-	2,594	15.93	91.71
100-	1,350	8.29	100

Notes: EQ-VAS: European Quality of Life – Visual Analogue Scale, Cum. Percentage – Cumulative percentage

For peer review only

Appendix 2: Relationship between EQ-VAS and EQ5D across major sub-groups

Dependent variable	Mobility	Self-care	Usual care	Pain / discomfort	Anxiety / Depression	Observation
Overall	-10.5 (0.6)	-9.9 (0.9)	-12.4 (0.7)	-10.9 (0.6)	-8.9 (0.8)	16287
Gender						
**Male**	-12.0 (0.6)	-11.4 (1.1)	-14.6 (0.9)	-11.9 (0.6)	-12.1 (0.7)	7760
**Female**	-8.1 (0.4)	-7.9 (0.7)	-9.9 (0.6)	-7.7 (0.4)	-9.1 (0.5)	8527
Age groups						
**Young (20-44 yrs)**	-9.9 (0.5)	-11.0 (0.9)	-12.0 (0.8)	-8.8 (0.5)	-9.3 (0.6)	9603
**Middle (45-60 yrs)**	-9.0 (0.5)	-10.8 (0.9)	-11.3 (0.8)	-9.0 (0.5)	-10.7 (0.6)	5544
**Elderly (>60 yrs)**	-11.1 (1.0)	-11.7 (1.6)	-13.1 (1.3)	-11.0 (1.1)	-13.2 (1.4)	1140
Income						
**Low income group (INR <10000 or US$ 155)**	-10.6 (0.4)	-12.1 (0.7)	-13.2 (0.6)	-10.2 (0.4)	-10.7 (0.5)	11537
**Middle income group (INR 10000-20000 or US$ 155-310)**	-10.2 (0.8)	-8.9 (1.6)	-10.7 (1.3)	-9.2 (0.8)	-11.8 (1.1)	2667
**High income group (INR >20000 or US$>310)**	-12 (1.0)	-14.4(2.5)	-15.6 (1.7)	-10.5 (0.9)	-8.2 (1.6)	1975
Level of education						
**Up to primary school**	-10.8 (0.6)	-12.8 (1.1)	-12.6 (0.8)	-9.9 (0.6)	-11.6 (0.8)	3604
**Secondary school**	-9.9 (0.4)	-11.1 (0.8)	-12.2 (0.7)	-9.3 (0.4)	-10.2 (0.5)	9924
**Graduation and above**	-8.9 (1.0)	-8.3 (2.1)	-11.4 (1.8)	-9.8 (0.9)	-8.3 (1.3)	2759
Marital status						
**Single**	-8.4 (1.9)	-9.4 (3.8)	-15.2 (3.3)	-9.0 (1.8)	-10.8 (2.0)	1177
**Married**	-10.3 (0.4)	-11.5 (0.6)	-12.3 (0.5)	-9.5 (0.4)	-10.3 (0.5)	14217
**Widowed**	-9.9 (1.2)	-11.1 (1.8)	-12.9 (1.4)	-10.1 (1.2)	-11.8 (1.5)	838
**Divorce**	-18.6 (5.8)	-14.1 (7.0)	-24.2 (6.0)	-15.1 (5.6)	-15.6 (5.1)	55
Tobacco use						
**No**	-10.1 (0.4)	-11.4 (0.6)	-12.4 (0.5)	-9.8 (0.4)	-10.5 (0.5)	12529
**Yes**	-13.4 (0.8)	-15.5 (1.5)	-16.5 (1.2)	-11.1 (0.7)	-12.6 (0.9)	3758
Alcohol use						
**No**	-10.6 (0.4)	-12.1 (0.6)	-13.1 (0.5)	-10.2 (0.4)	-11.0 (0.5)	13911
**Yes**	-10.6 (1.1)	-10.7 (2.0)	-11.4 (1.9)	-8.2 (1.0)	-10.6 (1.2)	2376
BMI						
**Underweight**	-10.6 (2.4)	-15.6 (5.1)	-15.4 (3.9)	-10.5 (2.9)	-7.8 (4.8)	756
**Normal weight**	-10.6 (0.9)	-11.6(1.5)	-13.6 (1.2)	-9.2 (0.9)	-9.7 (1.2)	5278
**Overweight**	-10.3 (1.1)	-12.1 (1.2)	-11.6 (1.3)	-7.7 (0.9))	-8.9 (1.2)	4190
**Obesity**	-7.4 (0.9)	-9.9 (1.7)	-11.4 (1.3)	-8.0 (0.9)	-8.6 (1.4)	2249
Diabetes						
**Diabetes (diagnosed or self-reported)**	-10.0 (0.6)	-11.6 (1.1)	-11.6 (0.9)	-9.7 (0.6)	-11.4 (0.8)	3676
**Pre diabetes**	-10.2 (0.6)	-13.5 (1.0)	-14.1 (0.8)	-9.4 (0.6)	-10.6 (0.7)	5449
**No diabetes**	-10.9 (0.7)	-11.0 (1.2)	-12.6 (1.1)	-9.9 (0.7)	-10.0 (0.8)	4610
Hypertension						

For peer review only - http://bmjopen.bmj.com/site/about/guidelines.xhtml

**Diagnosed or Self-reported hypertension**	-11.2 (0.6)	-14.7 (1.0)	-14.1 (0.8)	-10.8 (0.6)	-10.8 (0.7)	5074
**Pre hypertension**	-10.9 (0.7)	-12.4 (1.2)	-14.1 (1.0)	-9.7 (0.7)	-11.2 (0.9)	4717
**No hypertension**	-9.3 (0.6)	-9.6 (1.0)	-10.4 (0.9)	-8.4 (0.6)	-10.0 (0.7)	5695
Heart disease						
**No**	-10.5 (0.3)	-11.9 (0.6)	-13.0 (0.5)	-9.9 (0.3)	-10.7 (0.4)	15842
**Yes**	-9.0 (1.6)	-9.0 (2.5)	-9.5 (1.9)	-7.4 (1.7)	-11.8 (2.0)	445
Stroke						
**No**	-10.7 (0.3)	-11.9 (0.6)	-13.1 (0.5)	-10.0 (0.3)	-10.8 (0.4)	16203
**Yes**	-13.7 (3.6)	-18.4 (4.4)	-19.2 (4.1)	-12.2 (3.5)	-16.3 (4.1)	84
Kidney disease						
**No**	-10.7 (0.3)	-11.9 (0.6)	-13.1 (0.5)	-9.9 (0.3)	-10.8 (0.4)	16175
**Yes**	-11.6 (3.7)	-21.2 (4.8)	-14.3 (4.2)	-16.4 (3.2)	-16.3 (3.7)	112

NOTES: Tobacco use, Heart disease, Kidney disease were based on self-reports, newly diagnosed diabetes - defined as no self-reported diabetes and fasting blood glucose (FBG) of ≥126 mg/dl, or HbA1c ≥6.5%), pre-diabetes – no self-reported diabetes and FBG ≥100-125 mg/dl or HbA1c ≥5.7-6.4%), normoglycemia – no self-reported diabetes and FBG<100 mg/dl and HbA1c<5.7%, Newly diagnosed hypertension – defined as no self-reported hypertension and BP ≥140/90mmHg, prehypertension - no self-reported hypertension and BP: 120-139 / 80-89 mmHg and normotensive - no history of hypertension and BP <120/80 mmHg. INR: Indian rupees, mmHg – millimeter of mercury, mg/dl - Milligram/deciliter; yrs – years; US$ - United States Dollar

For peer review only

For peer review only - http://bmjopen.bmj.com/site/about/guidelines.xhtml

Appendix 3 - The comparison of HRQOL as measured by EQ5D-VAS among different countries

**Countries, year of study**	**N**	**EQ-VAS**	**Percentages with any difficulties in EQ5D domain**					
		Mean	Mobility	Self-care	Usual activities	Pain / Discomfort	Anxiety / Depression	Any dimension
India, **Delhi (age≥20), 2011**	5,365	78.9	14.1	1.6	4.4	8.0	9.5	27.8
India, **Chennai, (age≥20), 2011**	6,903	70.8	17.3	7.8	7.7	9.0	8.9	29.7
Pakistan **(Karachi), (age≥20), 2011**	4,016	73.2	10.4	1.9	5.6	8.7	9.3	17.9
China **(age≥18), 2008**	2,991	77.0	4.9	2.0	3.3	18.0	6.1	22.4
UK **(age≥18), 1998**	3395	82.5	18.4	4.2	16.3	33.0	20.9	43.1
USA **(age≥18) , 1998**	427	82.2	14.0	3.0	14.0	40.0	24.0	na
Japan **(age≥20) , 1998**	620	77.8	7.2	1.8	5.2	20.0	8.5	25.0
Spain **(age≥15) , 1998**	12,245	71.1	11.2	2.0	6.9	26.3	12.5	33.0
Canada **(age≥18), 1997**	1518	78.7	22.2	4.0	19.1	43.6	28.6	53.0
Sweden **(age≥18), 1998**	3069	83.5	10.0	2.0	8.0	42.0	30.0	na
Finland **(age≥18), 1992**	2411	79.4	20.0	5.0	18.0	39.0	14.0	na
Germany **(age≥18), 1998**	337	82.2	18.0	3.0	13.0	37.0	18.0	na
Belgium **(age≥18), 2001**	1274	81.0	13.0	3.0	15.0	42.0	21.0	na
New Zealand **(age≥18), 1999**	1328	81.3	17.0	4.0	18.0	37.0	20.0	na
Zimbabwe **(age≥18), 2000**	2350	76.1	20.0	7.0	18.0	41.0	40.0	na
Armenia **(age≥18), 2002**	2222	66.6	26.0	13.0	28.0	64.0	52.0	na

NOTES: EQ5D-VAS: European Quality of Life 5 Dimension – Visual Analogue Scale; na – not available

For peer review only - http://bmjopen.bmj.com/site/about/guidelines.xhtml

STROBE 2007 (v4) Statement—Checklist of items that should be included in reports of *cross-sectional studies*

Section/Topic	Item #	Recommendation	Reported on page #
**Title and abstract**	1	(*a*) Indicate the study's design with a commonly used term in the title or the abstract	1, 6
		(*b*) Provide in the abstract an informative and balanced summary of what was done and what was found	6
**Introduction**			
Background/rationale	2	Explain the scientific background and rationale for the investigation being reported	8,9
Objectives	3	State specific objectives, including any prespecified hypotheses	9
**Methods**			
Study design	4	Present key elements of study design early in the paper	9
Setting	5	Describe the setting, locations, and relevant dates, including periods of recruitment, exposure, follow-up, and data collection	9
Participants	6	(*a*) Give the eligibility criteria, and the sources and methods of selection of participants	9, 10
Variables	7	Clearly define all outcomes, exposures, predictors, potential confounders, and effect modifiers. Give diagnostic criteria, if applicable	10
Data sources/ measurement	8*	For each variable of interest, give sources of data and details of methods of assessment (measurement). Describe comparability of assessment methods if there is more than one group	10, 11
Bias	9	Describe any efforts to address potential sources of bias	*12 (adjusted regression model was run to adjust for potential confounders)*
Study size	10	Explain how the study size was arrived at	Not applicable – secondary data analysis
Quantitative variables	11	Explain how quantitative variables were handled in the analyses. If applicable, describe which groupings were chosen and why	12

For peer review only

Statistical methods	12	(*a*) Describe all statistical methods, including those used to control for confounding	12
		(*b*) Describe any methods used to examine subgroups and interactions	Not applicable
		(*c*) Explain how missing data were addressed	Not applicable
		(*d*) If applicable, describe analytical methods taking account of sampling strategy	Not applicable
		(*e*) Describe any sensitivity analyses	Not applicable
**Results**			
Participants	13*	(a) Report numbers of individuals at each stage of study—eg numbers potentially eligible, examined for eligibility, confirmed eligible, included in the study, completing follow-up, and analysed	13
		(b) Give reasons for non-participation at each stage	Not applicable
		(c) Consider use of a flow diagram	Not applicable
Descriptive data	14*	(a) Give characteristics of study participants (eg demographic, clinical, social) and information on exposures and potential confounders	13
		(b) Indicate number of participants with missing data for each variable of interest	13
Outcome data	15*	Report numbers of outcome events or summary measures	13
Main results	16	(*a*) Give unadjusted estimates and, if applicable, confounder-adjusted estimates and their precision (eg, 95% confidence interval). Make clear which confounders were adjusted for and why they were included	13
		(*b*) Report category boundaries when continuous variables were categorized	13
		(*c*) If relevant, consider translating estimates of relative risk into absolute risk for a meaningful time period	Not applicable
Other analyses	17	Report other analyses done—eg analyses of subgroups and interactions, and sensitivity analyses	13 - 15
**Discussion**			
Key results	18	Summarise key results with reference to study objectives	15
Limitations	19	Discuss limitations of the study, taking into account sources of potential bias or imprecision. Discuss both direction and magnitude of any potential bias	19
Interpretation	20	Give a cautious overall interpretation of results considering objectives, limitations, multiplicity of analyses, results from similar studies, and other relevant evidence	16-18
Generalisability	21	Discuss the generalisability (external validity) of the study results	18 - 19
**Other information**			
Funding	22	Give the source of funding and the role of the funders for the present study and, if applicable, for the original study on which the present article is based	20

For peer review only

*Give information separately for cases and controls in case-control studies and, if applicable, for exposed and unexposed groups in cohort and cross-sectional studies.

Note: An Explanation and Elaboration article discusses each checklist item and gives methodological background and published examples of transparent reporting. The STROBE checklist is best used in conjunction with this article (freely available on the Web sites of PLoS Medicine at http://www.plosmedicine.org/, Annals of Internal Medicine at http://www.annals.org/, and Epidemiology at http://www.epidem.com/). Information on the STROBE Initiative is available at www.strobe-statement.org.

For peer review only
